# A Retrospective Analysis Reveals That the 2021 Outbreaks of African Swine Fever Virus in Ghana Were Caused by Two Distinct Genotypes

**DOI:** 10.3390/v16081265

**Published:** 2024-08-07

**Authors:** Ayushi Rai, Edward Spinard, Jehadi Osei-Bonsu, Amanda Meyers, Mark Dinhobl, Vivian O’Donnell, Patrick T. Ababio, Daniel Tawiah-Yingar, Daniel Arthur, Daniel Baah, Elizabeth Ramirez-Medina, Nallely Espinoza, Alyssa Valladares, Bonto Faburay, Aruna Ambagala, Theophilus Odoom, Manuel V. Borca, Douglas P. Gladue

**Affiliations:** 1 U.S. Department of Agriculture, Agricultural Research Service, Foreign Animal Disease Research Unit, Plum Island Animal Disease Center, Orient, NY 11957, USA; ayushi.rai@usda.gov (A.R.); edward.spinard@usda.gov (E.S.); amanda.meyers@usda.gov (A.M.); mark.dinhobl@usda.gov (M.D.); elizabeth.ramirez@usda.gov (E.R.-M.); nallely.espinoza@usda.gov (N.E.); alyssa.valladares@usda.gov (A.V.); 2 Oak Ridge Institute for Science and Education (ORISE), Oak Ridge, TN 37830, USA; 3 U.S. Department of Agriculture, Agricultural Research Service, Foreign Animal Disease Research Unit, National Bio and Agro-Defense Facility, Manhattan, KS 66502, USA; 4 Accra Veterinary Laboratory of Veterinary Services Directorate, Accra P.O. Box GA184, Ghana; jehdvets@gmail.com (J.O.-B.); ginolapaatee@gmail.com (P.T.A.); nanayawtee@icloud.com (D.T.-Y.); danielarthur42681@gmail.com (D.A.); dannyneph@gmail.com (D.B.); theodoom@yahoo.com (T.O.); 5 Department of Liberal Arts & Sciences, University of Illinois at Urbana-Champaign, Champaign, IL 61820, USA; 6 U.S. Department of Agriculture, Animal and Plant Inspection Service, Plum Island Animal Disease Center, Greenport, NY 11944, USA; vivian.odonnell@usda.gov; 7 U.S. Department of Agriculture, Animal and Plant Inspection Service, National Bio and Agro-Defense Facility, Greenport, NY 11944, USA; bonto.faburay@usda.gov; 8 National Centre for Foreign Animal Disease, Canadian Food Inspection Agency, Winnipeg, MB R3E 3M4, Canada; aruna.ambagala@inspection.gc.ca

**Keywords:** African swine fever, ASFV, Ghana, genome

## Abstract

African swine fever virus (ASFV) is the causative agent of African swine fever (ASF), a highly infectious and lethal disease of domesticated swine. Outbreaks of ASF have been mostly restricted to the continent of Africa. The outbreaks that have occurred outside of Africa were controlled by extensive depopulation of the domesticated pig population. However, in 2007, an outbreak occurred in the country of Georgia, where ASFV infected wild pigs and quickly spread across eastern Europe. Since the reintroduction of ASF into Europe, variants of the current pandemic strain, ASFV Georgia 2007/01 (ASFV-G), which is classified as Genotype 2 based on p72 sequencing, have been reported in countries within western Europe, Asia, and the island of Hispaniola. Additionally, isolates collected in 2020 confirmed the presence of variants of ASFV-G in Nigeria. Recently, we reported similar variants of ASFV-G collected from domestic pigs suspected of dying of ASF in Ghana in 2022. Here, we retroactively report, based on full-length sequencing, that similar variants were present in Ghana in 2021. The SNP analysis revealed derivatives of ASFV with distinct genetic markers. Furthermore, we identified three full-length ASFV genomes as Genotype 1, indicating that there were two genotypes circulating in proximity during the 2021 ASF outbreaks in Ghana.

## 1. Introduction

African swine fever (ASF) is a highly contagious and fatal viral disease of domestic and wild pigs. Its first reported occurrence was in Kenya in 1921, and since then, it has caused numerous outbreaks across different regions of Africa [1]. Although sporadic outbreaks have been reported outside of Africa, they have been contained and controlled via mass culling and by restricting the transport of susceptible animals and animal products. However, in 2007, an outbreak in the Republic of Georgia quickly spread to neighboring countries and after an introduction into China in 2018, which resulted in the rapid spread of ASF across all of southeastern Asia [2]. Concurrently, the disease continued to spread in both eastern and central Europe and has been causing continued outbreaks as far west as Germany, where recently, ASF outbreaks occurred not only in wild boar but also in pig farms. In 2021, the first outbreak in the western hemisphere occurred in the Dominican Republic, and outbreaks have continued since on the island of Hispaniola; this was the first time the island of Hispaniola had ASF outbreaks since the late 1970s which was only resolved by the culling of all swine on the island [1,3,4].

The etiological agent of the disease, African swine fever virus (ASFV), is an icosahedral, enveloped virus belonging to the *Asfivirus* genus of the *Asfarviridae* family. The virus’s genome is composed of double-stranded DNA that varies in size from 170 to 192 kilobases and encodes between 150 and 200 open reading frames (ORFs). The functions of the majority of genes have not been experimentally verified; rather, some of the gene functions have been predicated based on sequence and structure homology [5,6]. The diversity observed in ASFV is largely attributed to the gain, loss, truncation, elongation, and fusion of proteins classified within one of the five multigene families (MGF): MGF-100, MGF-110, MGF-300, MGF-360, and MGF-505 [7,8,9,10]. Moreover, structural prediction suggests that MGF genes within a family can have unique functions [6,11]. It is noteworthy that virulent and attenuated [12] isolates harboring deletions of MGF genes have been sequenced, emphasizing the complex involvement of these genes in ASFV virulence [13,14,15].

Historically, due to sequencing limitations, ASFV was classified into 25 genotypes based on the partial sequencing of B646L, which encodes for the major capsid protein p72 [16,17]. Yet, the number of differences that defined a genotype was never established and led to establishment of genotypes at will. Accordingly, ASFV was recently reclassified into five distinct genotypes based on a maximum allowance of two amino acid differences [18], and a tool for easy analysis was developed [19]. Other ASFV classification systems have attempted to include p54 and the central variable region (CVR) of B602L with p72 [20]. Still, the complexity of the ASFV genome renders classifications based on a limited number of genes inadequate, and we developed a method for Biotyping [21] based on all the open reading frames. Further, recombination events, such as the one observed in China that resulted in the generation of isolates composed of sequences originating from NHV (Genotype 1) and ASFV-G (Genotype 2), would have remained unreported [22]. Accordingly, complete genome sequencing can enhance comprehension of the number of circulating ASFV genomes and is of vital importance to understand and track the continued evolution of ASFV strains.

Between 1996 and 1997, the western African countries of Cote D’Ivoire, Togo, Benin, and Nigeria reported outbreaks of ASF [12]. Shortly thereafter, the first reported outbreak of ASF in Ghana occurred in the fall of 1999 [12]. Previous studies have reported that Genotype 1 was the predominant p72 genotype responsible for outbreaks of ASF in Ghana [23]. However, we recently demonstrated that Genotype 2 was responsible for the 2022 outbreaks of domestic swine in Ghana [24]. Here, we examined the genomes of 13 isolates collected from nine different regions experiencing outbreaks in 2021. Ten of the genomes were highly similar to the Genotype 2 isolates sequenced from Ghana in 2022, indicating that derivatives of the current pandemic strain emerged in Ghana earlier than reported. Still, the SNP analysis revealed synonymous and nonsynonymous mutations, including large deletions leading to fusions within proteins of the MGF 360 family, indicating the continued evolution of ASFV and the existence of unique Genotype 2 variants. Furthermore, three of the isolates were demonstrated to be Genotype 1 and were more similar to the Genotype 1 isolate sequenced from Benin in 1997 (Benin 97/1 and AM712239) than to the Genotype 1 genomes sequenced from Europe in the 1960s–1970s (E75, Ourt 88/3, NHV, and L60). The data indicate that Genotype 1 was still causing outbreaks in Ghana during this sampling period. Comparatively, the Genotype 1 isolates sequenced were more diverse than the Genotype 2 isolates, suggesting potential extended evolution of Genotype 1 isolates in Ghana, as expected from previous reports of Genotype 1 in Ghana, where Genotype 2 was likely recently introduced and thus less diverse during the sampling period. These findings are significant, as they expand our understanding of the epidemiology of ASF in Ghana and highlight the need for the continued surveillance and monitoring of ASFV genotypes in domestic swine populations, especially since outbreaks of Genotypes 1 and 2 are occurring in proximity, which could lead to a potential recombination event, as was recently observed in Asia.

## 2. Materials and Methods

### 2.1. Next-Generation Sequencing

ASFV isolates were passed once in blood derived primary swine macrophage cultures produced as previously described [25]. Viral genomes were sequenced as previously described by using an Illumina Nextseq500 sequencing platform [26]. To summarize, viral DNA was extracted from the cytoplasmic fraction of infected macrophage cultures by using a nuclear extraction kit (Active Motif, Carlsbad, CA, USA). For the creation of libraries, the Nextera XT kit (Illumnia, San Diego, CA, USA) was utilized in accordance with the manufacturer’s instructions. Furthermore, to bridge across low-complexity regions of the genome, Ghana2021-95 was sequenced by using the Oxford nanopore GridION sequencing platform with previously described methods [24].

### 2.2. Genome Assembly and SNP Detection

All steps were performed by using QIAGEN CLC Genomics Workbench 23.0 (QIAGEN, Aarhus, Denmark). Illumina reads were trimmed for quality (limit = 0.05), ambiguous base pairs (max = 2), adapters, and minimum size (min = 50) and from the 5′ (20 nucleotides) and 3′ terminal end (5 nucleotides). GridION reads were not trimmed for quality. To construct the genome of Ghana2021-95, 2,152,588 GridION reads and 270,361,688 (paired) and 83,058 (orphan) trimmed Illumina reads were assembled by using the default parameters of the “De Novo Assembly Long Reads and Polish with Short Reads” pipeline. Following assembly, the contig corresponding to ASFV was extracted, and all Illumina reads were mapped back to the extracted contig, resulting in an average depth of coverage of 1601 reads. The consensus sequence was extracted, resulting in a 184,940 nt genome. To construct the genomes of Ghana2021-01 and Ghana2022-75, Illumina reads originating from each sample were trimmed as previously described and separately mapped to the Ghana2021-95 genome, and the consensus sequences were extracted. To construct the genomes of Ghana2021-13, Ghana2021-27, Ghana2021-37, Ghana2021-41, Ghana2021-49, Ghana2021-57, Ghana2021-81, Ghana2021-87, Ghana2021-91, and Ghana2021-105, Illumina reads originating from each sample were trimmed as previously described and separately mapped to the Ghana2022-35 genome (OP479889) [24], and the consensus sequences were extracted. To identify Single-Nucleotide Polymorphisms (SNPs), the 2021 Ghana genomes were mapped to their indicated reference by using the Map Long Reads to Reference module with long-read splice alignment disabled, followed by the Basic Variant Detection and Amino Acid Changes modules.

### 2.3. Annotation of Genome

All annotations were performed by using Genome Annotation Transfer Utility [27]. By using Ghana2022-35 as a reference, annotations were transferred to Ghana2021-13, Ghana2021-27, Ghana2021-37, Ghana2021-41, Ghana2021-49, Ghana2021-57, Ghana2021-81, Ghana2021-87, Ghana2021-91, and Ghana2021-105. Annotations were transferred to Ghana2021-95 by using Benin 97/1 (AM712239) as a reference. Annotations were transferred to Ghana2021-01 and Ghana2021-75 by using Ghana2021-95 as a reference.

### 2.4. Genome Alignment

A previously described database of genomes consisting of 32 ASFV genomes [24] was aligned against the 2021 and 2022 sequences originating from Ghana by using the Create Whole-Genome Alignment module by CLC Genomics, and a phylogeny was constructed by using four combinations of the comparisons Average Nucleotide Identity (ANI) or Average Alignment Percentage (AP) and the methods Neighbor Joining (NJ) or Unweighted Pair Group Method with Arithmetic Mean (UPGMA).

### 2.5. Protein Alignment

The protein translations from all annotated ORFs originating from the genomes isolated from Ghana were compared against the NCBI database by using the default parameters of BLASTP. Proteins containing unique sequences were collected along with homologs encoded by the Ghana 2022 isolates and a previously described list of historical isolates [24] and were aligned by using MUSCLE [28], using the default parameters Gap Open = 10 and Gap extension = 1.

## 3. Results

### 3.1. Characteristics of Collected 2021 Outbreak Samples

**Figure 1:**
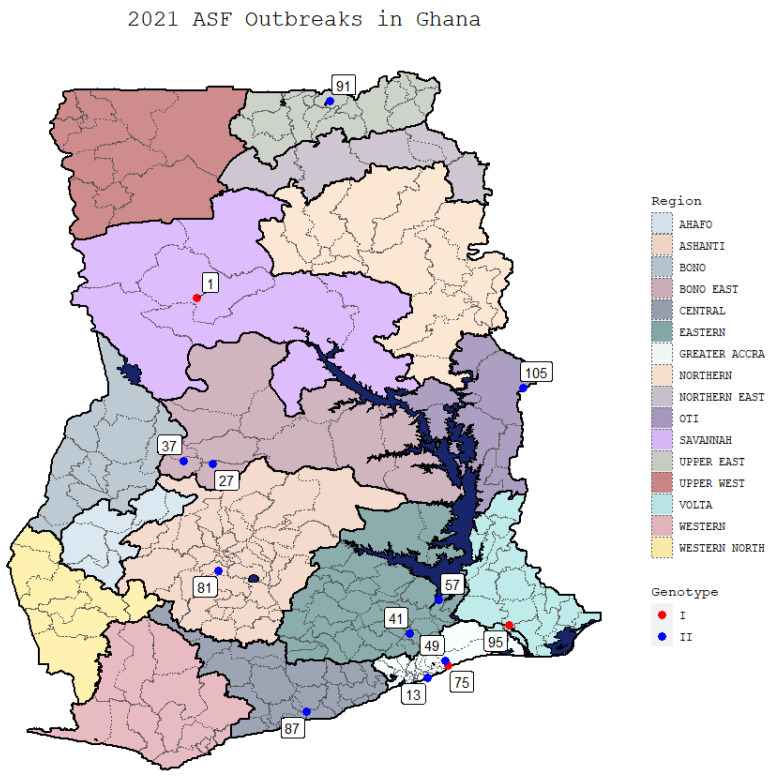
A map of the different regions in Ghana with the indicated outbreaks identified. The different p72 genotypes are differentiated by red (Genotype 1) or blue (Genotype 2) circles. Sample 1 from the Savannah region was collected on 5-1-2021; Sample 13 from the Greater Accra region was collected on 17-3-2021; Sample 27 from the Bono East region was collected on 3-3-2021; Sample 37 from the Bono East region was collected on 20-9-2021; Sample 41 from the Eastern region was collected on 9-10-2021; Sample 49 from the Greater Accra region was collected on 17-11-2021; Sample 57 from the Eastern region was collected on 17-11-2021; Sample 75 from the Greater Accra region was collected on 17-7-2021; Sample 81 from the Ashanti region was collected on 23-4-2021; Sample 87 from the Central region was collected on 3-9-2021; Sample 91 from the Upper East region was collected on 8 6 2021; Sample 95 from the Volta region was collected on 10-11-2021; Sample 105 from the Oti region was collected on 11-8-2021.

Thirteen isolates of ASFV were obtained from outbreaks during 2021, from the Savannah, Greater Accra, Bono East, Eastern, Central East, Volta, and Oti regions of Ghana (Figure 1 and Table 1) on the indicated dates, from farms with suspect ASF outbreaks.

### 3.2. Genotyping of ASFV Isolates

Genotyping was performed on all thirteen isolates. Ghana2022-01, Ghana2022-75, and Ghana2022-95 were determined to be Genotype 1 based on the coding sequences of p72 (B646L) (REF) (Table 1). The remaining isolates (Ghana2021-01, Ghana2021-13, Ghana2021-27, Ghana2021-37, Ghana2021-41, Ghana2021-49, Ghana2021-57, Ghana2021-75, Ghana2021-81, Ghana2021-87, Ghana2021-91, and Ghana2021-105) were determined to be Genotype 2 (Table 1).

### 3.3. ASFV Full-Genome Alignments

**Figure 2:**
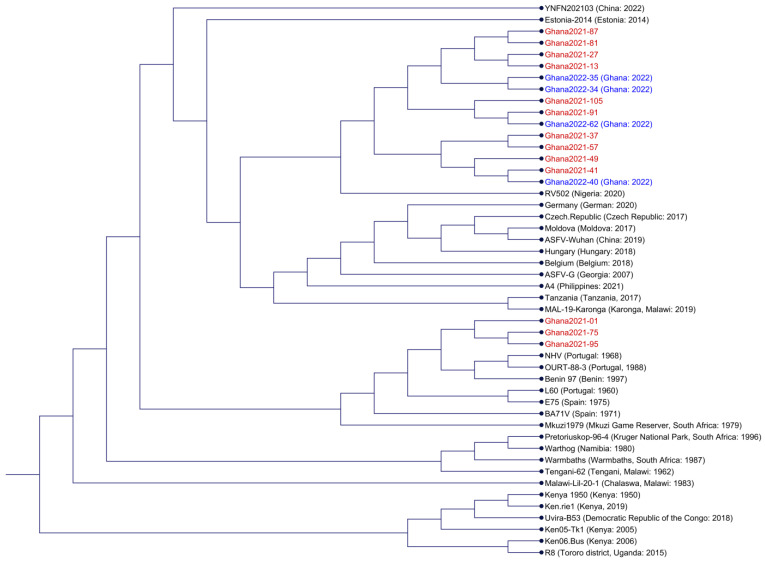
Cladogram of ASFV isolates using ANI and NJ. Ghana genomes are highlighted in red (2021) and blue (2022) fonts.

To gain a broad understanding of how the Ghana2021 genomes compared to historic and recent ASFV isolates, whole-genome alignment was performed with representative isolates of different genotypes (Figure 2). Genotype 2 isolates were chosen for containing large deletions and fusions of MGF genes (YFN202103, Estonia2014, and RV502) and for originating from eastern (Mal-19-Karonga, Tengain-62, and Tanzania), southern (Pretoriuskop/96/4, Warmbaths, and Warthog) and western (Ghana2022-34, Ghana2022-25, and Ghana2022-62) Africa, Asia (A4 and ASFV-Wuhan), and Europe (German, Czech Republic, Maldova, Hungary, Belgium, and ASFV-G). Genotype 1 isolates were chosen for being attenuated (NHV and OURT-88/3), for being a lab-type strain (BA71V), and for originating from Europe (E75 and L60) and Africa (Benin 97 and Mkuzi 1979). Genotype 8 was represented by Malawi-Lil-20-1, and Genotype 9 was represented by Kenya1950, Ken.rie1, Uvira-B53, Ken05.Tk1, Ken06-Bus, and R8.

Not surprisingly, the Genotype 2 Ghana2021 isolates (Ghana2021-13, Ghana2021-27, Ghana2021-37, Ghana2021-41, Ghana2021-49, Ghana2021-57, Ghana2021-81, Ghana2021-87, Ghana2021-91, and Ghana2021-105) formed a major clade along with the Ghana2022 isolates that were previously determined to belong to p72 Genotype 2. The Ghana 2021-2022 clade formed a sister group with Nigeria RV502, which was previously demonstrated to share a sister group with the Ghana2022 isolate [15,24]. Like the isolates from 2022, the 2021 isolates do not contain the reverse complement duplication of the 5′ end of the genome at the 3′ end of the genome that was observed in the RV502 genome. The Ghana2021-22/RV502 clade shared a sister group with a clade that is composed two clades: the European/Asian Genotype 2 clade and the eastern African Genotype 2 clade. Based on whole-genome alignment, Estonia 2014 and YFN202103 were determined to be Genotype 2 outliers.

**Figure 3:**
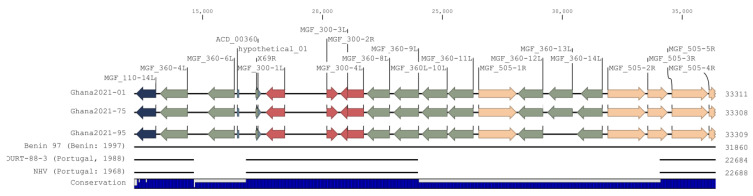
Genome alignment of Ghana p72 Genotype 1 isolates (Ghana2021-01, Ghana2021-75, and Ghana2021-95), Benin 97 (AM712239.1), OURT-88-3 (AM712240.1), and NHV (KM262845.1). Annotated MGF_110 (blue), MGF_360 (green), 224vMGF_300 (red), and MGF_505 (peach) family genes are indicated by colored arrows. Deletions are represented by the absence of a black line. The royal blue bar graph beneath the figure indicates nucleotide conservation.

Genotype 1 isolates (Ghana2021-01, Ghana2021-75, and Ghana2021-95) formed a clade that was a sister group to the clade composed of Benin 97, NHV, and OURT-88/3. However, unlike NHV and OURT-88-3, further examination of the genomes between nucleotides positions 10,000 and 35,000 demonstrates that Benin 97, Ghana2021-01, Ghana2021-75, and Ghana2021-95 do not contain deletions of MGF_360-6L, MGF_360-10 to 14L, and MGF_505-1R to -3R (Figure 3). The deletion of these MGF genes is believed to contribute to the attenuation of virulence observed in NHV and OURT-88-3 [5,29,30,31], and this deletion was not observed in the Ghana Genotype 1 isolates. A super clade composed of all Genotype 1 isolates (the previously mentioned isolates, E75, L60, BA71V, and Mkuzi1979) is a sister group to the Genotype 2 super clade. Taken together, based on whole-genome alignment analysis, the Ghana2021 genomes are the most similar to genomes of the same genotype that were collected from the same region.

### 3.4. Genetic Variation among Ghana 2021 Genotype 2 Isolates

**Figure 4:**
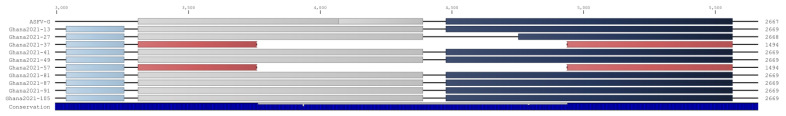
Genome alignment of LVR (nucleotides positions ~3000 to ~5600) of ASFV-G and Genotype 2 Ghana 2021 isolates. ORFs are indicated by colored rectangles as follows: KP93L (light blue); MGF_360-1L, MGF_360-1La, and MGF_3601Lb (light grey); MGF_360-2L (dark blue); and MGF_360-1L-2L fusion (red). Deletions are represented by the absence of a black line. The royal blue bar graph beneath the figure indicates nucleotide conservation.

To better understand the different groupings observed in the cladogram, the genomes of the 2021 Ghana isolates were compared to a reference sequence. The Genotype 2 genomes (Ghana2021-13, Ghana2021-27, Ghana2021-37, Ghana2021-41, Ghana2021-49, Ghana2021-57, Ghana2021-81, Ghana2021-87, Ghana2021-91, and Ghana2021-105) were compared to Ghana2022-35, the first outbreak strain in Ghana identified in 2022 (Table S1). The total number of SNPs and the number of nonsynonymous SNPs per genome that were detected in the Ghana2021 genomes are summarized in Table 2. The encoded protein sequence of ORFs containing nonsynonymous mutations were submitted to BLASTP on NCBI [32,33,34,35]. Sequences that did not have a match of 100% identity and 100% coverage were considered to be a unique protein sequence specific to the 2021 Ghana genomes and were aligned to historic isolates (Figure S1). The specific mutations, compared with the indicated reference sequence, are summarized in Table S2. Ghana2021-13 had 13 SNPs compared with Ghana2022-35 (Table 3), of which 5 were nonsynonymous, resulting in three unique protein sequences: NP419L (P17S), MGF_360-10L (E238D), and MGF_360-13L (ATSTK262-266QHQPS and an insertion of YAFST between amino acids at position 283–285). Ghana2021-27 contained 15 SNPs compared with Ghana2022-35 (Table 3), of which 7 were nonsynonymous, resulting in four unique protein sequences: A137R (S107N), E199L (E125V), G1340L (R91H), and a frameshift in MGF_360-2L that resulted in a 92-amino acid truncation (Figure 4). Ghana2021-37 contained 20 SNPs compared with Ghana2022-35 (Table 3), of which 5 were nonsynonymous, resulting in two unique protein sequences: K145R (Y116H) and a 1174 nt deletion that resulted in in-frame fusion between MGF_360-1L and MGF_360-2L (Figure 4). Ghana2021-41 contained 22 SNPs compared with Ghana2022-35 (Table 3), of which 7 were non synonymous, resulting in four unique protein sequences: C315R (Q30H), MGF_505-1R (R450I), and the previously mentioned mutations in K145R and MGF_360-13L. Ghana2021-49 contained 23 SNPs compared with Ghana2022-35 (Table 3), of which 8 were nonsynonymous, resulting in four unique protein sequences: E146L (G39E) and the previously mentioned mutations of C315R, K145R, and MGF_360-13L. Ghana2021-57 contained 20 SNPs compared with Ghana2022-35 (Table 3), of which 8 were nonsynonymous, resulting in five unique protein sequences: C717R (K613N), P1192R (E39G), the previously mentioned fusion of MGF_360-1L and -2L, and the previously mentioned mutations in K145R and MGF_360-13L. Ghana2021-81 contained five SNPs compared with Ghana2022-35 (Table 3), of which three were nonsynonymous, resulting in one unique protein sequence, C717R, which was same as the mutation observed in Ghana2021-57. Ghana2021-87 contained five SNPs compared with Ghana2022-35 (Table 3), of which three were nonsynonymous, yet resulting in zero unique protein sequences. Ghana2021-91 contained 10 SNPs compared with Ghana2022-35 (Table 3), of which 6 were nonsynonymous, resulting in two unique protein sequences: C475L (Q148H) and the previously mentioned mutation in MGF_360-13L. Lastly, Ghana2021-105 contained 15 SNPs compared with Ghana2022-35 (Table 3), of which 9 were nonsynonymous, resulting in two unique protein sequences: C717R (K609T and EK612-613QN) and the previously mentioned mutation in MGF_360-13L. In summary, the Ghana2021 Genotype 2 genomes had on average 14.8 (±6.26) SNPs, which resulted, on average, in a change in the coding sequence of 6.1 (±1.97) ORFs per genome. The data indicate the continued evolution of ASFV Genotype 2 genomes in Africa.

### 3.5. Genetic Variation among Ghana 2021 Genotype 1 Isolates

Here, we report the first fully sequenced Genotype 1 isolate from Ghana, Ghana2021-95, which was compared to the historical isolate Benin 97/1 (Table S1). Subsequently, Ghana2021-01 and Ghana2021-75 were compared to Ghana2021-95. Compared with Benin 97/1, Ghana2021-95 had 63 SNPs, 25 of which were nonsynonymous, resulting in 16 unique protein sequences: a 254-amino acid truncation of A589L, B169L (A152T), B407L (an insertion of GSIRN between the amino acids at positions 121 and 122), B602L (a deletion of the first 77 amino acids), C84L (S12L), E11R (A35T), E183L (a deletion of amino acids at positions 106 to 110), E199L (T20I), E301R (M231T), EP364R (A343V), F1055L (V120I), MGF_360-15R (insertion and modification of amino acids of ECYST---------Y--304-309VLQHILERKFNILGLIP), MGF_360-1L (H58R), MGF_505-9R (E83K), NP1450L (P220L), and NP868R (L446F) (Table 4). Ghana2021-01 contained 44 SNPs compared with Ghana2021-95, of which 18 were nonsynonymous, resulting in 12 unique protein sequences: MGF_360-14L (mutations and deletions of AADFDRAQLIAHKAYMYNLSNIFLVKQLFSRDVTLVLDVTEPQEIYDMLKTYTSKNMKRAEEYLTAHPEI 284-353 GPPILIG-------------------------------------------------------HNSLRTKL, V355C, and D357T), MGF_360-19R (mutations and deletions of ANINQAM269-275G------), and the previously mentioned mutations in B407L, B602L, C84L, E111R, E301R, F1055L, MGF_360-16R, and NP1450L (Table 4). Ghana2021-75 contained 43 SNPs compared with Ghana2021-95, of which 15 were nonsynonymous, resulting in 12 unique proteins: MGF_360-6L (G94S) and the previously described mutations in B407L, B602L, C84L, E111R, E183L, E199L, E301R, F1055L, MGF_360-16R, MGF_360-19R, and NP1450L (Table 4). Of note, when Ghana2021-01 was aligned to Ghana2021-75, a total of 42 SNPs were observed. (In summary, the Ghana2021 Genotype 1 genomes had on average 43.5 (±0.50) SNPs, which resulted, on average, in a change in coding sequence of 19.33 (±1.50) ORFs per genome. The data indicate that there is more variation in the Genotype 1 Ghana 2021 genomes compared with the Genotype 2 genomes. Further, the Ghana2021 Genotype 1 genomes contain markers that differentiate them from Benin 97/1.

## 4. Discussion

Here, we report thirteen full-length sequences obtained from the ASF outbreaks that occurred in Ghana in 2021. The data indicate that variants of the 2007 Genotype 2 pandemic strain were in Ghana a year earlier than previously believed. Furthermore, our findings identified the complete genomes of three Genotype 1 isolates that exhibited sequence homology to Benin 97/1, suggesting that some outbreaks can still be attributed to Genotype 1 strains, which historically have been the cause of ASF outbreaks in Ghana [23]. The cladistics analysis revealed that the 2021 Genotype 1 strains share a common ancestor and that the Genotype 2 strains share a common ancestor. Further, the examination of the ORFs revealed several distinct mutations that are common among the Ghana 2021 Genotype 1 and Genotype 2 isolates. Still, the SNP analysis identified unique differences among the genomes, indicating the continued evolution of ASFV in Ghana; although this study had limited sampling and it is possible that there were other circulating strains in Ghana, it provides the first body of evidence that in Ghana, there were two distinct circulating strains causing outbreaks in the same time period. Both the Genotype 1 and 2 strains came from swine from suspected cases of ASFV.

As reviewed by Njau et al., based on the full or partial sequencing of p72, 12 different genotypes based on the historic classification (I, II, III, IX, X, XI, XII, XIII, XIV, XV, XVIII, and XXII) or the recent reclassification (1, 2, 8, and 9) have been responsible for ASF outbreaks in Africa [17]. The full-genome analysis revealed that the Genotype 2 isolates collected from Tanzania and Malawi are unique enough to form a separate clade compared with the clade composed of European and Asian Genotype 2 isolates (Figure 2) and the clade composed of Genotype 2 isolates collected from Nigeria and Ghana [36]. Consequently, while the partial sequencing of p72 provides a general comprehension of the ongoing outbreaks, it is imperative to continue complete genome sequencing and analysis to ascertain the specific variants of ASFV responsible for the outbreaks in Africa.

Whole-genome sequencing is essential when looking at ASFV outbreaks. Recently, a hybrid Genotype 1 and 2 strain was observed in China [22], possibly due to incorrect vaccine matching and outbreak strains. This strain with p72 would have not been identified correctly if not for the full-genome sequencing of ASFV. As ASFV vaccines become commercially available [37], understating the full genomes of ASFV causing outbreaks is essential in order to be able to predict protection with ASFV vaccines.

## Figures and Tables

**Table 1:** Origin and genotyping of Ghana samples.

Isolate	GenBank Accession	Region	Date of Outbreak	Genotype (p72)
Ghana2021-01	OR371517	Savannah	5-1-2021	1
Ghana2021-13	OR371519	Greater Accra	17-3-2021	2
Ghana2021-27	OR371520	Bono East	3-3-2021	2
Ghana2021-37	OR371521	Bono East	20-9-2021	2
Ghana2021-41	OR371522	Eastern	9-10-2021	2
Ghana2021-49	OR371523	Greater Accra	17-11-2021	2
Ghana2021-57	OR371524	Eastern	17-11-2021	2
Ghana2021-75	OR371525	Greater Accra	17-7-2021	1
Ghana2021-81	OR371526	Ashanti	23-4-2021	2
Ghana2021-87	OR371527	Central	3-9-2021	2
Ghana2021-91	OR371528	Upper East	8-6-2021	2
Ghana2021-95	OR371529	Volta	10-11-2021	1
Ghana2021-105	OR371518	Oti	11-8-2021	2

**Table 2:** Summary of the identified SNPs in the Ghana 2021 genomes.

Genotype	Isolate	Reference	Total SNPs Compared with Reference	Total Nonsynonymous SNPs Compared with Reference
2	Ghana2021-13	Ghana2022-35	13	5
	Ghana2021-27	Ghana2022-35	15	7
	Ghana2021-37	Ghana2022-35	20	5
	Ghana2021-41	Ghana2022-35	22	7
	Ghana2021-49	Ghana2022-35	23	8
	Ghana2021-57	Ghana2022-35	20	8
	Ghana2021-81	Ghana2022-35	5	3
	Ghana2021-87	Ghana2022-35	5	3
	Ghana2021-91	Ghana2022-35	10	6
	Ghana2021-105	Ghana2022-35	15	9
1	Ghana2021-95	Benin 97/1	63	25
	Ghana2021-01	Ghana2021-95	44	18
	Ghana2021-75	Ghana2021-95	43	15

**Table 3:** Unique protein sequences encoded by Ghana2021 Genotype 2 genomes.

	Ghana2021-13	Ghana2021-27	Ghana2021-37	Ghana2021-41	Ghana2021-49	Ghana2021-57	Ghana2021-81	Ghana2021-87	Ghana2021-91	Ghana2021-105
A137R		S107N								
C315R				Q30H	Q30H					
C475L									Q148H	Q148H
C717R						K613N	K613N			K609T, EK612-613QN
E146L					G39E					
E199L		E125V								
G1340L		R91H								
K145R			Y116H	Y116H	Y116H	Y116H				
MGF 360-10L	E238D									
MGF 360-13L	ATSTK262-266QHQPS, Ins283-283YAFST			ATSTK262-266QHQPS, Ins283-283YAFST	ATSTK262-266QHQPS, Ins283-283YAFST	ATSTK262-266QHQPS, Ins283-283YAFST			ATSTK262-266QHQPS, Ins283-283YAFST	ATSTK262-266QHQPS, Ins283-283YAFST
MGF 360-1L			fusion			fusion				
MGF 360-2L		truncation	fusion			fusion				
MGF 505-1R				R450I						
NP419L	P17S									
P1192R						E39G				

Footer: Ins = insert.

**Table 4:** Unique protein sequences encoded by Ghana2021 Genotype 1 genomes.

	Ghana2021-75	Ghana2021-95	Ghana2021-01
A859L		Truncation	
B169L		A152T	
B407L	Ins121-121GSIRN	Ins121-121GSIRN	Ins121-121GSIRN
B602L	MAEFNIDELLKNVLEDPSTEISEETLKQLYQRTNPYKQFKNDSRVAFCSFTNLREQYIRRLIMTSFIGYVFKALQEW1-77Del	MAEFNIDELLKNVLEDPSTEISEETLKQLYQRTNPYKQFKNDSRVAFCSFTNLREQYIRRLIMTSFIGYVFKALQEW1-77Del	MAEFNIDELLKNVLEDPSTEISEETLKQLYQRTNPYKQFKNDSRVAFCSFTNLREQYIRRLIMTSFIGYVFKALQEW1-77Del
C475L			P329L
C84L	S12L	S12L	S12L
E111R	A35T	A35T	A35T
E183L	RPATN106-110Del	RPATN106-110Del	RPATN106-110Del
E199L	T20I	T20I	
E301R	M231T	M231T	M231T
EP364R		A343V	
F1055L	V120I	V120I	V120I
MGF_360-14L			AADFDRAQLIAHKAYMYNLSNIFLVKQLFSRDVTLVLDVTEPQEIYDMLKTYTSKNMKRAEEYLTAHPEI284-353GPPILIG-------------------------------------------------------HNSLRTKL, V355C, D357T
MGF_360-16R	ECYST----------Y--304-309VLQHIILERKNIPLGLFL	ECYST---------Y--304-309VLQHILERKFNILGLIP	ECYST---------Y--304-309VLQHILERKFNILGLIP
MGF_360-19R	ANINQAM269-275G------	Na	ANINQAM269-275G------
MGF_360-1L		H58R	
MGF_360-6L	G94S		
MGF_505-9R		E283K	
NP1450L	P220L	P220L	P220L
NP868R		L466F	

Footer: Ins = insert. Na = not annotated. Del = deletion.

## Data Availability

Genome sequences have been deposited in GenBank under accession Nos. OR371517 (Ghana2021-01), OR371519 (Ghana2021-13), OR371520 (Ghana2021-27), OR371521 (Ghana2021-37), OR371522 (Ghana2021-41), OR371523 (Ghana2021-49), OR371524 (Ghana2021-57), OR371525 (Ghana2021-75), OR371526 (Ghana2021-81), OR371527 (Ghana2021-87), OR371528 (Ghana2021-91), OR371529 (Ghana2021-95), and OR371518 (Ghana2021-105). Raw data for this project can be found at BioProject under accession No. PRJNA894113.

## Supplementary Materials

The following supporting information can be downloaded at: https://www.mdpi.com/article/10.3390/v16081265/s1, Figure S1: Protein alignments; Table S1: All Ghana 2021 SNPs.
