# Supplementary material for: A Retrospective Analysis Reveals That the 2021 Outbreaks of African Swine Fever Virus in Ghana Were Caused by Two Distinct Genotypes
Source: Viruses. 2024 Aug 7;16(8):1265. doi: 10.3390/v16081265 (PMC11360390; doi:10.3390/v16081265)
Supplement: Supplementary file 1 [file viruses-16-01265-s001.zip › Figure S1 Protein alignments.zip!/AlignmentPDF/A137R.pdf]

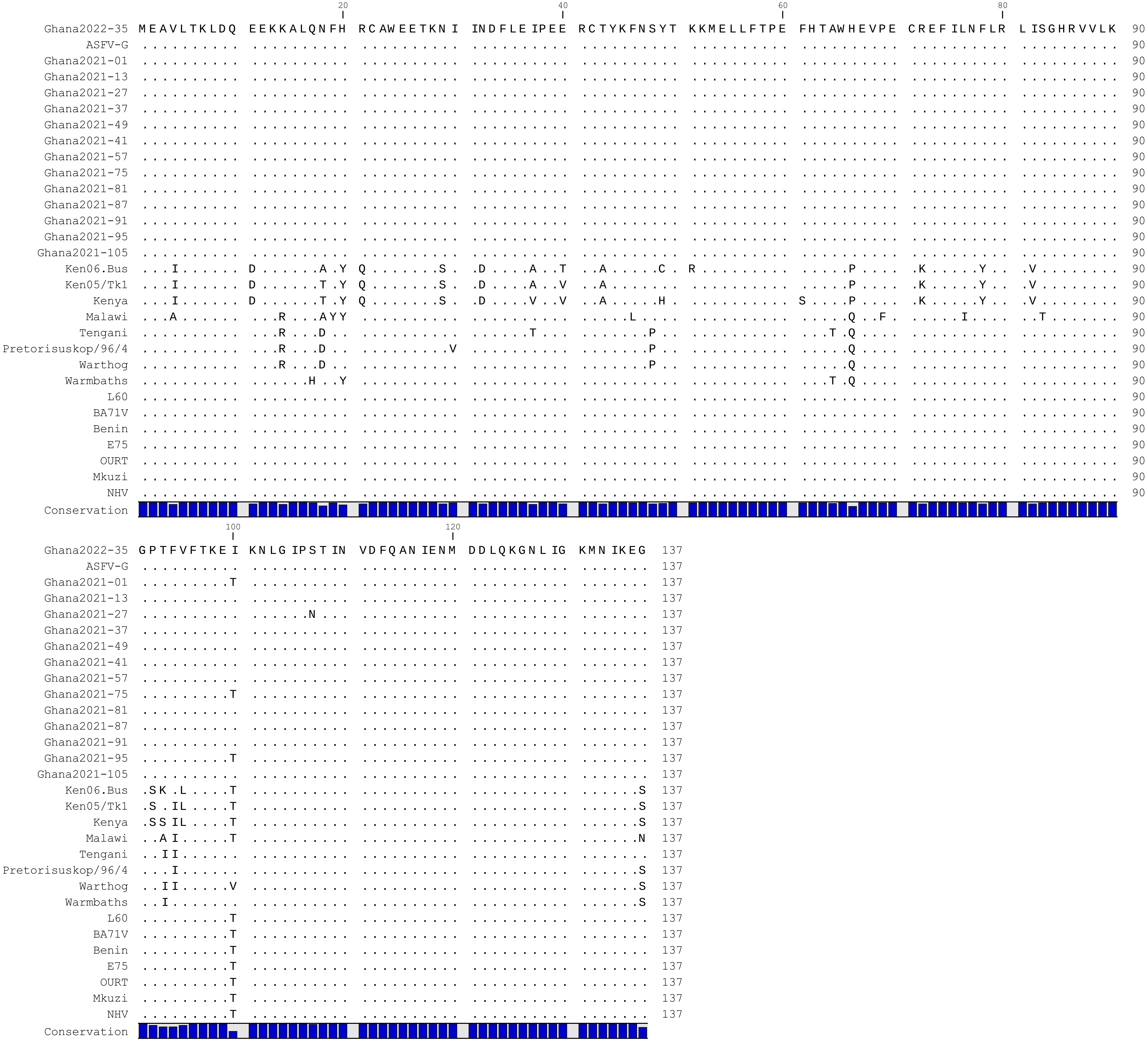
20
40
60
80
Ghana2022-35 MEAVLTKLDQ EEKKALQNFH RCAWEETKNI INDFLEIPEE RCTYKFNSYT KKMELLFTPE FHTAWHEVPE CREFILNFLR LISGHRVVLK 90
ASFV-G
Ghana2021-01
Ghana2021-13
Ghana2021-27
Ghana2021-37
Ghana2021-49
Ghana2021-41
Ghana2021-57
Ghana2021-75
Ghana2021-81
Ghana2021-87
Ghana2021-91
Ghana2021-95
Ghana2021-105
Ken06.Bus
Ken05/Tk1
Kenya
Malawi
Tengani
Pretorisuskop/96/4
Warthog
Warmbaths
L60
BA71V
Benin
E75
OURT
Mkuzi
NHV
Conservation
100
120
Ghana2022-35 GPTFVFTKEI KNLGIPSTIN VDFQANIENM DDLQKGNLIG KMNIKEG 137
Conservation
