# Supplementary material for: A Retrospective Analysis Reveals That the 2021 Outbreaks of African Swine Fever Virus in Ghana Were Caused by Two Distinct Genotypes
Source: Viruses. 2024 Aug 7;16(8):1265. doi: 10.3390/v16081265 (PMC11360390; doi:10.3390/v16081265)
Supplement: Supplementary file 1 [file viruses-16-01265-s001.zip › Figure S1 Protein alignments.zip!/AlignmentPDF/B169L.pdf]

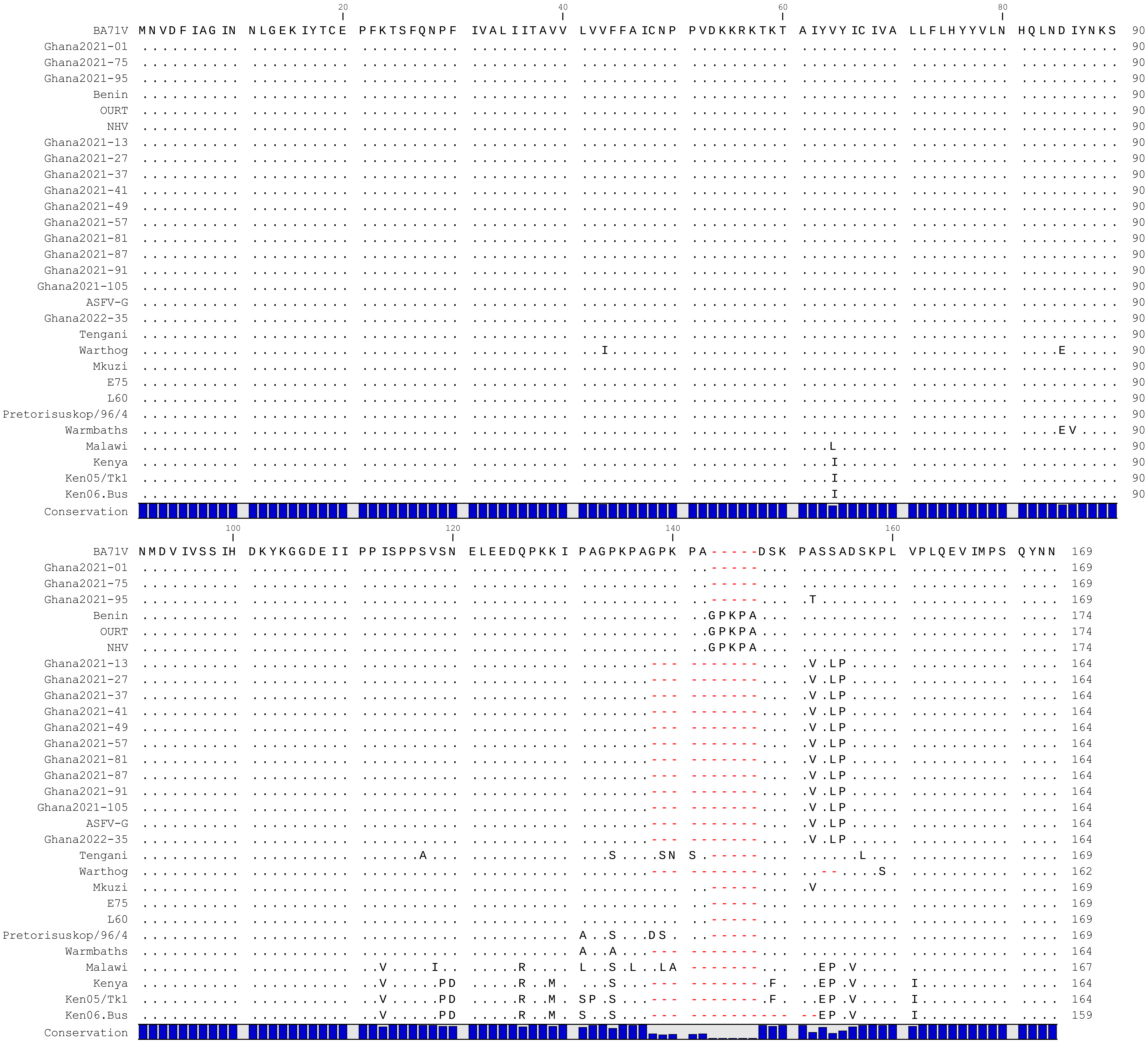
20 40 60 80
BA71V MNVDFIAGIN NLGEKIYTCE PFKTSFQNPF IVALIITAVV LVVFFAICNP PVDKKRKTKT AIYVYICIVA LLFLHYYVLN HQLNDIYNKS 90
Ghana2021-01 90
Ghana2021-75 90
Ghana2021-95 90
Benin 90
OURT 90
NHV 90
Ghana2021-13 90
Ghana2021-27 90
Ghana2021-37 90
Ghana2021-41 90
Ghana2021-49 90
Ghana2021-57 90
Ghana2021-81 90
Ghana2021-87 90
Ghana2021-91 90
Ghana2021-105 90
ASFV-G 90
Ghana2022-35 90
Tengani 90
Warthog ....I....... ....E..... 90
Mkuzi 90
E75 90
L60 90
Pretorisuskop/96/4 90
Warmbaths ....EV.... 90
Malawi ....L...... 90
Kenya ....I...... 90
Ken05/Tk1 ....I...... 90
Ken06.Bus ....I...... 90
Conservation
100 120 140 160
BA71V NMDVIVSSIH DKYKGGDEII PPISPPSVSN ELEEDQPKKI PAGPKPAGPK PA-----DSK PASSADSKPL VPLQEVIMPS QYNN 169
Ghana2021-01 ....-----... 169
Ghana2021-75 ....-----... 169
Ghana2021-95 ....-----... .T........ 169
Benin ....GPKPA... 174
OURT ....GPKPA... 174
NHV ....GPKPA... 174
Ghana2021-13 ....--- -------... .V.LP..... 164
Ghana2021-27 ....--- -------... .V.LP..... 164
Ghana2021-37 ....--- -------... .V.LP..... 164
Ghana2021-41 ....--- -------... .V.LP..... 164
Ghana2021-49 ....--- -------... .V.LP..... 164
Ghana2021-57 ....--- -------... .V.LP..... 164
Ghana2021-81 ....--- -------... .V.LP..... 164
Ghana2021-87 ....--- -------... .V.LP..... 164
Ghana2021-91 ....--- -------... .V.LP..... 164
Ghana2021-105 ....--- -------... .V.LP..... 164
ASFV-G ....--- -------... .V.LP..... 164
Ghana2022-35 ....--- -------... .V.LP..... 164
Tengani ....A... ....S....SN S.-----... ......L... 169
Warthog ....--- -------... ..--....S. 162
Mkuzi ....-----... .V........ 169
E75 ....-----... 169
L60 ....-----... 169
Pretorisuskop/96/4 .... A..S...DS. ..-----... 169
Warmbaths .... A..A...--- -------... 164
Malawi ....V....I.. .....R.... L..S.L..LA -------... ..EP.V.... 167
Kenya ....V.....PD .....R..M. ...S...--- -------.F. ..EP.V.... I......... 164
Ken05/Tk1 ....V.....PD .....R..M. SP.S...--- -------.F. ..EP.V.... I......... 164
Ken06.Bus ....V.....PD .....R..M. S..S...--- ---------- --EP.V.... I......... 159
Conservation
