# Supplementary material for: A Retrospective Analysis Reveals That the 2021 Outbreaks of African Swine Fever Virus in Ghana Were Caused by Two Distinct Genotypes
Source: Viruses. 2024 Aug 7;16(8):1265. doi: 10.3390/v16081265 (PMC11360390; doi:10.3390/v16081265)
Supplement: Supplementary file 1 [file viruses-16-01265-s001.zip › Figure S1 Protein alignments.zip!/AlignmentPDF/B407L.pdf]

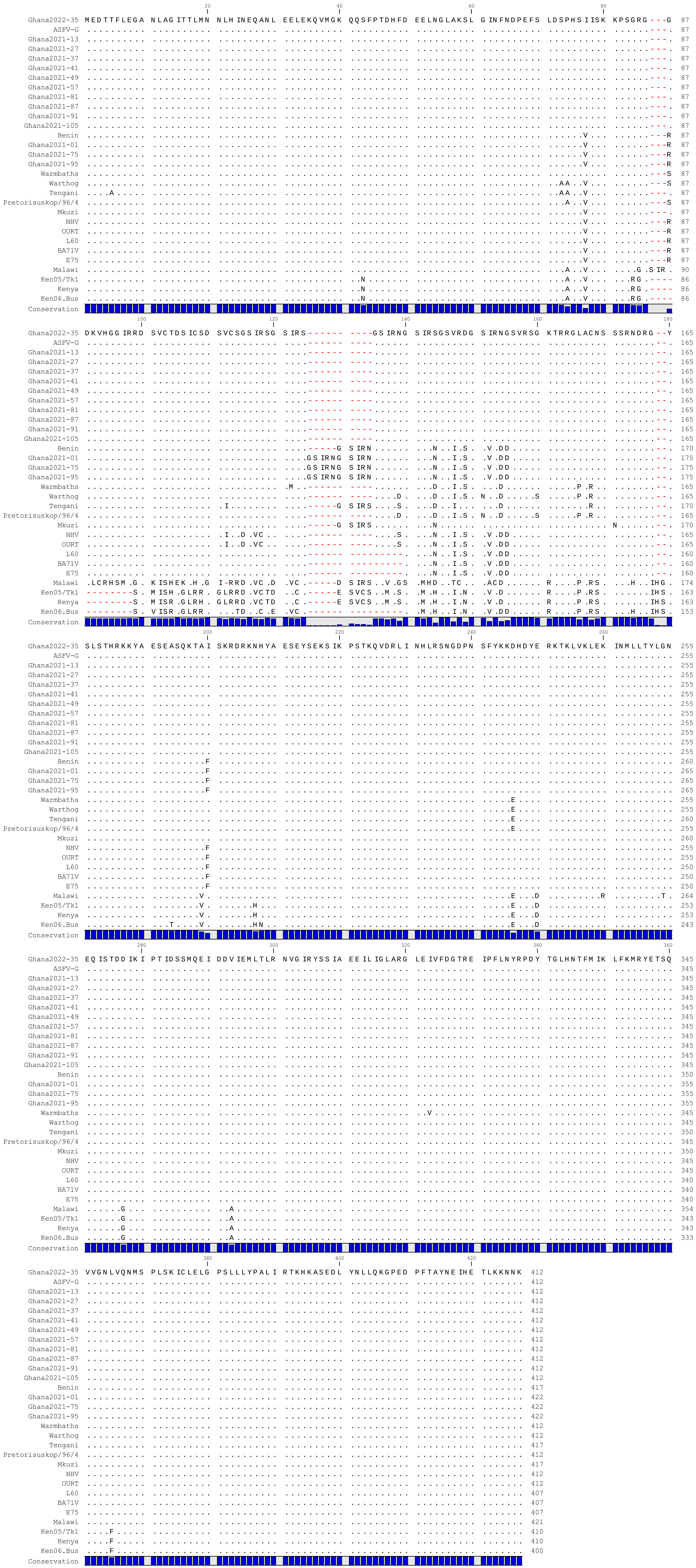
Ghana2022-35 MEDTTFLEGA NLAGITTLMN NLHINEQANL EELEKQVMGK QQSFPTDHFD EELNGLAKSL GINFNDPEFS LDSPHSIISK KPSGRG---G 87
Ghana2022-35 DKVHGGIRRD SVCTDSICSD SVCSGSIRSG SIRS------ ----GSIRNG SIRSGSVRDG SIRNGSVRSG KTRRGLACNS SSRNDRG--Y 165
Ghana2022-35 SLSTHRKKYA ESEASQKTAI SKRDRKNHYA ESEYSEKSIK PSTKQVDRLI NHLRSNGDPN SFYKKDHDYE RKTKLVKLEK INMLLTYLGN 255
Ghana2022-35 EQISTDDIKI PTIDSSMQEI DDVIEMLTLR NVGIRYSSIA EEILIGLARG LEIVFDGTRE IPFLNYRPDY TGLHNTFMIK LFKMRYETSQ 345
Ghana2022-35 VVGNLVQNMS PLSKICLELG PSLLLYPALI RTKHKASEDL YNLLQKGPED PFTAYNEIHE TLKKNNK 412
ASFV-G
Ghana2021-13
Ghana2021-27
Ghana2021-37
Ghana2021-41
Ghana2021-49
Ghana2021-57
Ghana2021-81
Ghana2021-87
Ghana2021-91
Ghana2021-105
Benin
Ghana2021-01
Ghana2021-75
Ghana2021-95
Warmbaths
Warthog
Tengani
Pretorisuskop/96/4
Mkuzi
NHV
OURT
L60
BA71V
E75
Malawi
Ken05/Tk1
Kenya
Ken06.Bus
Conservation
