# Supplementary material for: A Retrospective Analysis Reveals That the 2021 Outbreaks of African Swine Fever Virus in Ghana Were Caused by Two Distinct Genotypes
Source: Viruses. 2024 Aug 7;16(8):1265. doi: 10.3390/v16081265 (PMC11360390; doi:10.3390/v16081265)
Supplement: Supplementary file 1 [file viruses-16-01265-s001.zip › Figure S1 Protein alignments.zip!/AlignmentPDF/C315R.pdf]

```
                                         20                  40                  60                  80
Ghana2022-35        MDALLKEIEK LSQP-SLQKE NNDVCDLCFM QMKKISNYQL LCEECGQLKD WFEPEYNEKF TVYSRLKIVG ANSSYHQRDL DKANSSDYSS  89
ASFV-G              .......... ....-..... .......... .......... .......... .......... .......... .......... ..........  89
Ghana2021-13        .......... ....-..... .......... .......... .......... .......... .......... .......... ..........  89
Ghana2021-37        .......... ....-..... .......... .......... .......... .......... .......... .......... ..........  89
Ghana2021-27        .......... ....-..... .......... .......... .......... .......... .......... .......... ..........  89
Ghana2021-41        .......... ....-..... .......... H......... .......... .......... .......... .......... ..........  89
Ghana2021-49        .......... ....-..... .......... H......... .......... .......... .......... .......... ..........  89
Ghana2021-57        .......... ....-..... .......... .......... .......... .......... .......... .......... ..........  89
Ghana2021-81        .......... ....-..... .......... .......... .......... .......... .......... .......... ..........  89
Ghana2021-91        .......... ....-..... .......... .......... .......... .......... .......... .......... ..........  89
Ghana2021-87        .......... ....-..... .......... .......... .......... .......... .......... .......... ..........  89
Ghana2021-105       .......... ....-..... .......... .......... .......... .......... .......... .......... ..........  89
Benin               .......... ....-..... .......... .......... .......... .......... .......... .......... ..........  89
Ghana2021-01        .......... ....-..... .......... .......... .......... .......... .......... .......... ..........  89
Ghana2021-75        .......... ....-..... .......... .......... .......... .......... .......... .......... ..........  89
Ghana2021-95        .......... ....-..... .......... .......... .......... .......... .......... .......... ..........  89
Malawi              .......... ....-..... .......... .......... .......... .......... .......... .......... ..........  89
L60                 .......... ....-..... .......... .......... .......... .......... .......... .......... ..........  89
BA71V               .......... ....-..... .......... .......... .......... .......... .......... .......... ..........  89
E75                 .......... ....-..... .......... .......... .......... .......... .......... .......... ..........  89
OURT                .......... ....-..... .......... .......... .......... .......... .......... .......... ..........  89
Tengani             .......... ....-..... .......... .......... .......... .......... S......... .......... ..........  89
Mkuzi               .......... ....-..... .......... .......... .......... .......... .......... .......... ..........  89
NHV                 .......... ....-..... .......... .......... .......... .......... .......... .......... ..........  89
Warmbaths           .......... ....-..... .......... .......... .......... .......... .......... .......... ..........  89
Warthog             .......... ....-..... .......... .......... .......... .......... .......... .......... ..........  89
Pretorisuskop/96/4  .......... ....-..... .......... .......... .......... .......... .......... .......... ..........  89
Ken06.Bus           .......... ....S..... .......... .......... .......... ....D..... .......... .......... ..........  90
Kenya               .......... ....S..... .......... .......... .......... ....D..... .......... .......... ..........  90
Ken05/Tk1           .......... ....S..... .......... .......... .......... ....D..... .......... .......... ..........  90
Conservation

                             100                 120                 140                 160                 180
Ghana2022-35        LQFHHILEEL KSLNVKYMDA GQKPFPIQVL KETAHSYNQV QQHRVIRSIT KLQILASILR SICLKLNIAC TVADAARFTQ LNTKGISRGM  179
ASFV-G              .......... .......... .......... .......... .......... .......... .......... .......... ..........  179
Ghana2021-13        .......... .......... .......... .......... .......... .......... .......... .......... ..........  179
Ghana2021-37        .......... .......... .......... .......... .......... .......... .......... .......... ..........  179
Ghana2021-27        .......... .......... .......... .......... .......... .......... .......... .......... ..........  179
Ghana2021-41        .......... .......... .......... .......... .......... .......... .......... .......... ..........  179
Ghana2021-49        .......... .......... .......... .......... .......... .......... .......... .......... ..........  179
Ghana2021-57        .......... .......... .......... .......... .......... .......... .......... .......... ..........  179
Ghana2021-81        .......... .......... .......... .......... .......... .......... .......... .......... ..........  179
Ghana2021-91        .......... .......... .......... .......... .......... .......... .......... .......... ..........  179
Ghana2021-87        .......... .......... .......... .......... .......... .......... .......... .......... ..........  179
Ghana2021-105       .......... .......... .......... .......... .......... .......... .......... .......... ..........  179
Benin               .......... .......... .......... .......... .......... .......... .......... .......... ..........  179
Ghana2021-01        .......... .......... .......... .......... .......... .......... .......... .......... ..........  179
Ghana2021-75        .......... .......... .......... .......... .......... .......... .......... .......... ..........  179
Ghana2021-95        .......... .......... .......... .......... .......... .......... .......... .......... ..........  179
Malawi              .......... .......... .......... .......... .......... .......... .......... .......... .......K..  179
L60                 .......... .......... .......... .......... .......... .......... .......... .......... ..........  179
BA71V               .......... .......... .......... .......... .......... .......... .......... .......... ..........  179
E75                 .......... .......... .......... .......... .......... .......... .......... .......... ..........  179
OURT                .......... .......... .......... .......... .......... .......... .......... .......... ..........  179
Tengani             .......... .......... .......... .......... .......... .......... .......... .......... ..........  179
Mkuzi               .......... .......... .......... .......... .......... .......... .......... .......... ..........  179
NHV                 .......... .......... .......... .......... .......... .......... .......... .......... ..........  179
Warmbaths           .......... .T........ .......... .......... .......... .......... .......... .......... ..........  179
Warthog             .......... .T........ .......... .......... .......... .......... .......... .......... ..........  179
Pretorisuskop/96/4  .......... .......... .......... .......... .......... .......... .......... .......... ..........  179
Ken06.Bus           .......... .......... .......... .......... .......... .......... .......... .......... ..........  180
Kenya               .......... .......... .......... .......... .......... .......... .......... .......... ..........  180
Ken05/Tk1           .......... .......... .......... .......... .......... .......... .......... .......... ..........  180
Conservation

                                         200                 220                 240                 260
Ghana2022-35        DLLRSLFVDN KITLNVDLNP IDSFINSTYS ALQIKQIHQE LQEENVYNLK EIVKSFILYA DEKNIGVDLN RRTVVIATMY NVLRRAYYPI  269
ASFV-G              .......... .......... .......... .......... .......... .......... .......... .......... ..........  269
Ghana2021-13        .......... .......... .......... .......... .......... .......... .......... .......... ..........  269
Ghana2021-37        .......... .......... .......... .......... .......... .......... .......... .......... ..........  269
Ghana2021-27        .......... .......... .......... .......... .......... .......... .......... .......... ..........  269
Ghana2021-41        .......... .......... .......... .......... .......... .......... .......... .......... ..........  269
Ghana2021-49        .......... .......... .......... .......... .......... .......... .......... .......... ..........  269
Ghana2021-57        .......... .......... .......... .......... .......... .......... .......... .......... ..........  269
Ghana2021-81        .......... .......... .......... .......... .......... .......... .......... .......... ..........  269
Ghana2021-91        .......... .......... .......... .......... .......... .......... .......... .......... ..........  269
Ghana2021-87        .......... .......... .......... .......... .......... .......... .......... .......... ..........  269
Ghana2021-105       .......... .......... .......... .......... .......... .......... .......... .......... ..........  269
Benin               .......... .......... .......... .......... .......... .......... .......... .......... ..........  269
Ghana2021-01        .......... .......... .......... .......... .......... .......... .......... .......... ..........  269
Ghana2021-75        .......... .......... .......... .......... .......... .......... .......... .......... ..........  269
Ghana2021-95        .......... .......... .......... .......... .......... .......... .......... .......... ..........  269
Malawi              .......... .......... .......... .......... .......... .......... .......... .......... ..........  269
L60                 .......... .......... .......... .......... .......... .......... .......... .......... ..........  269
BA71V               .......... .......... .......... .......... .......... .......... .......... .......... ..........  269
E75                 .......... .......... .......... .......... .......... .......... .......... .......... ..........  269
OURT                .......... .......... .......... .......... .......... .......... .......... .......... ..........  269
Tengani             .......... .......... .......... .......... .......... .......... .......... .......... ..........  269
Mkuzi               .......... .......... .......... .......... .......... .......... .......... .......... ..........  269
NHV                 .......... .......... .......... .......... .......... .......... .......... .......... ..........  269
Warmbaths           .......... .......... .........N .......... .......... .......... .......... .......... ..........  269
Warthog             .......... .......... .........N .......... .......... .......... .......... .......... ..........  269
Pretorisuskop/96/4  .......... .......... .........N .......... .......... .......... .......... .......... ..........  269
Ken06.Bus           .......E.. .......... .......... .......... .......... .......V.. .......... .......... ..........  270
Kenya               .......E.. .......... .......... .......... .......... .......V.. .......... .......... ..........  270
Ken05/Tk1           .......E.. .......... .......... .......... .......... .......V.. .......... .......... ..........  270
Conservation

                             280                 300
Ghana2022-35        EIDTVVYQCK IRKNTITRAL KMYEDYYSHF KSLYEQYHLN AAKKLI  315
ASFV-G              .......... .......... .......... .......... ......  315
Ghana2021-13        .......... .......... .......... .......... ......  315
Ghana2021-37        .......... .......... .......... .......... ......  315
Ghana2021-27        .......... .......... .......... .......... ......  315
Ghana2021-41        .......... .......... .......... .......... ......  315
Ghana2021-49        .......... .......... .......... .......... ......  315
Ghana2021-57        .......... .......... .......... .......... ......  315
Ghana2021-81        .......... .......... .......... .......... ......  315
Ghana2021-91        .......... .......... .......... .......... ......  315
Ghana2021-87        .......... .......... .......... .......... ......  315
Ghana2021-105       .......... .......... .......... .......... ......  315
Benin               .......... .......... .......... .......... ......  315
Ghana2021-01        .......... .......... .......... .......... ......  315
Ghana2021-75        .......... .......... .......... .......... ......  315
Ghana2021-95        .......... .......... .......... .......... ......  315
Malawi              .......... .......... .......... .......... ......  315
L60                 .......... .......... .......... .......... ......  315
BA71V               .......... .......... .......... .......... ......  315
E75                 .......... .......... .......... .......... ......  315
OURT                .......... .......... .......... .......... ......  315
Tengani             .......... .......... .......... .......... ......  315
Mkuzi               .......... .......... .......... .......... ......  315
NHV                 .......... .......... .......... .......... ......  315
Warmbaths           .......... .......... .......... .......... ......  315
Warthog             .......... .......... .......... .......... ......  315
Pretorisuskop/96/4  .......... .......... .......... .......... ......  315
Ken06.Bus           .......... .......... .......... .......Q.. ......  316
Kenya               .......... .......... .......... .......Q.. ......  316
Ken05/Tk1           .......... .......... .......... .......Q.. ......  316
Conservation
```
