# Supplementary material for: A Retrospective Analysis Reveals That the 2021 Outbreaks of African Swine Fever Virus in Ghana Were Caused by Two Distinct Genotypes
Source: Viruses. 2024 Aug 7;16(8):1265. doi: 10.3390/v16081265 (PMC11360390; doi:10.3390/v16081265)
Supplement: Supplementary file 1 [file viruses-16-01265-s001.zip › Figure S1 Protein alignments.zip!/AlignmentPDF/C475L.pdf]

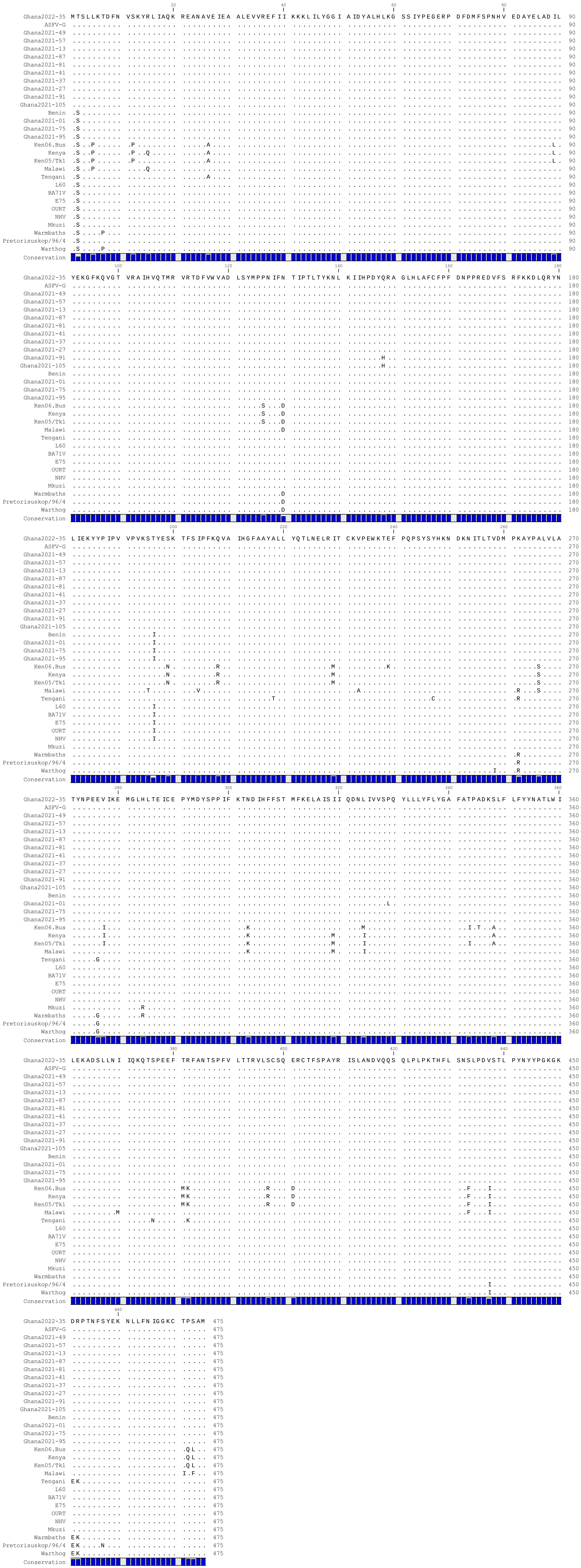
Ghana2022-35
ASFV-G
Ghana2021-49
Ghana2021-57
Ghana2021-13
Ghana2021-87
Ghana2021-81
Ghana2021-41
Ghana2021-37
Ghana2021-27
Ghana2021-91
Ghana2021-105
Benin
Ghana2021-01
Ghana2021-75
Ghana2021-95
Ken06.Bus
Kenya
Ken05/Tk1
Malawi
Tengani
L60
BA71V
E75
OURT
NHV
Mkuzi
Warmbaths
Pretorisuskop/96/4
Warthog
Conservation
MTSLLKTDFN VSKYRLIAQK REANAVEIEA ALEVVREFII KKKLILYGGI AIDYALHLKG SSIYPEGERP DFDMFSPNHV EDAYELADIL 90
YEKGFKQVGT VRAIHVQTMR VRTDFVWVAD LSYMPPNIFN TIPTLTYKNL KIIHPDYQRA GLHLAFCFPF DNPPREDVFS RFKKDLQRYN 180
LIEKYYPIPV VPVKSTYESK TFSIPFKQVA IHGFAAYALL YQTLNELRIT CKVPEWKTEF PQPSYSYHKN DKNITLTVDM PKAYPALVLA 270
TYNPEEVIKE MGLHLTEICE PYMDYSPPIF KTNDIHFFST MFKELAISII QDNLIVVSPQ YLLLYFLYGA FATPADKSLF LFYYNATLWI 360
LEKADSLLNI IQKQTSPEEF TRFANTSPFV LTTRVLSCSQ ERCTFSPAYR ISLANDVQQS QLPLPKTHFL SNSLPDVSTL PYNYYPGKGK 450
DRPTNFSYEK NLLFNIGGKC TPSAM 475
