# Supplementary material for: A Retrospective Analysis Reveals That the 2021 Outbreaks of African Swine Fever Virus in Ghana Were Caused by Two Distinct Genotypes
Source: Viruses. 2024 Aug 7;16(8):1265. doi: 10.3390/v16081265 (PMC11360390; doi:10.3390/v16081265)
Supplement: Supplementary file 1 [file viruses-16-01265-s001.zip › Figure S1 Protein alignments.zip!/AlignmentPDF/C84L.pdf]

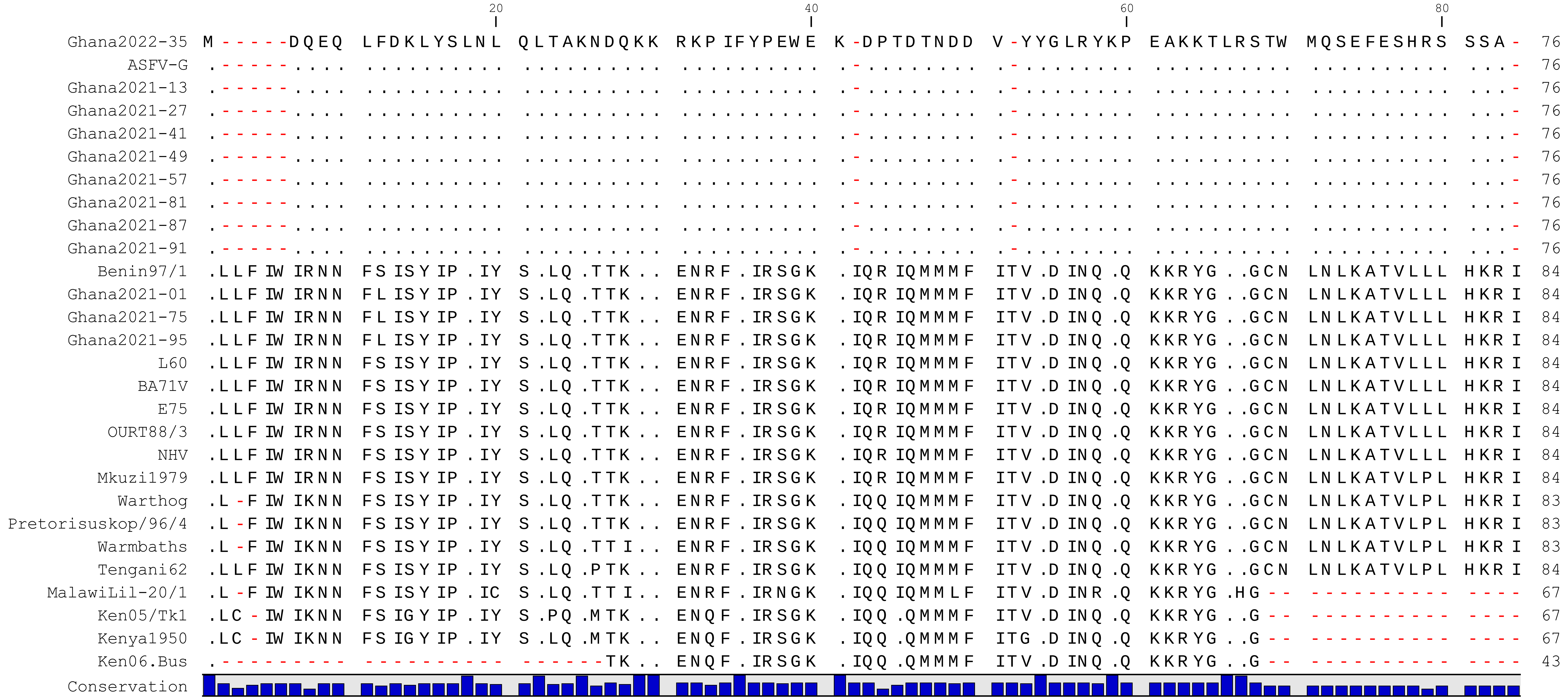
20 40 60 80
Ghana2022-35 M-----DQEQ LFDKLYSLNL QLTAKNDQKK RKPIFYPEWE K-DPTDTNDD V-YYGLRYKP EAKKTLRSTW MQSEFESHRS SSA- 76
ASFV-G .-----.... .... -......... .-........ ....- 76
Ghana2021-13 .-----.... .... -......... .-........ ....- 76
Ghana2021-27 .-----.... .... -......... .-........ ....- 76
Ghana2021-41 .-----.... .... -......... .-........ ....- 76
Ghana2021-49 .-----.... .... -......... .-........ ....- 76
Ghana2021-57 .-----.... .... -......... .-........ ....- 76
Ghana2021-81 .-----.... .... -......... .-........ ....- 76
Ghana2021-87 .-----.... .... -......... .-........ ....- 76
Ghana2021-91 .-----.... .... -......... .-........ ....- 76
Benin97/1 .LLFIWIRNN FSISYIP.IY S.LQ.TTK.. ENRF.IRSGK .IQRIQMMMF ITV.DINQ.Q KKRYG..GCN LNLKATVLLL HKRI 84
Ghana2021-01 .LLFIWIRNN FLISYIP.IY S.LQ.TTK.. ENRF.IRSGK .IQRIQMMMF ITV.DINQ.Q KKRYG..GCN LNLKATVLLL HKRI 84
Ghana2021-75 .LLFIWIRNN FLISYIP.IY S.LQ.TTK.. ENRF.IRSGK .IQRIQMMMF ITV.DINQ.Q KKRYG..GCN LNLKATVLLL HKRI 84
Ghana2021-95 .LLFIWIRNN FLISYIP.IY S.LQ.TTK.. ENRF.IRSGK .IQRIQMMMF ITV.DINQ.Q KKRYG..GCN LNLKATVLLL HKRI 84
L60 .LLFIWIRNN FSISYIP.IY S.LQ.TTK.. ENRF.IRSGK .IQRIQMMMF ITV.DINQ.Q KKRYG..GCN LNLKATVLLL HKRI 84
BA71V .LLFIWIRNN FSISYIP.IY S.LQ.TTK.. ENRF.IRSGK .IQRIQMMMF ITV.DINQ.Q KKRYG..GCN LNLKATVLLL HKRI 84
E75 .LLFIWIRNN FSISYIP.IY S.LQ.TTK.. ENRF.IRSGK .IQRIQMMMF ITV.DINQ.Q KKRYG..GCN LNLKATVLLL HKRI 84
OURT88/3 .LLFIWIRNN FSISYIP.IY S.LQ.TTK.. ENRF.IRSGK .IQRIQMMMF ITV.DINQ.Q KKRYG..GCN LNLKATVLLL HKRI 84
NHV .LLFIWIRNN FSISYIP.IY S.LQ.TTK.. ENRF.IRSGK .IQRIQMMMF ITV.DINQ.Q KKRYG..GCN LNLKATVLLL HKRI 84
Mkuzi1979 .LLFIWIRNN FSISYIP.IY S.LQ.TTK.. ENRF.IRSGK .IQRIQMMMF ITV.DINQ.Q KKRYG..GCN LNLKATVLPL HKRI 84
Warthog .L-FIWIKNN FSISYIP.IY S.LQ.TTK.. ENRF.IRSGK .IQQIQMMMF ITV.DINQ.Q KKRYG..GCN LNLKATVLPL HKRI 83
Pretorisuskop/96/4 .L-FIWIKNN FSISYIP.IY S.LQ.TTK.. ENRF.IRSGK .IQQIQMMMF ITV.DINQ.Q KKRYG..GCN LNLKATVLPL HKRI 83
Warmbaths .L-FIWIKNN FSISYIP.IY S.LQ.TTI.. ENRF.IRSGK .IQQIQMMMF ITV.DINQ.Q KKRYG..GCN LNLKATVLPL HKRI 83
Tengani62 .LLFIWIKNN FSISYIP.IY S.LQ.PTK.. ENRF.IRSGK .IQQIQMMMF ITV.DINQ.Q KKRYG..GCN LNLKATVLPL HKRI 84
MalawiLil-20/1 .L-FIWIKNN FSISYIP.IC S.LQ.TTI.. ENRF.IRNGK .IQQIQMMLF ITV.DINR.Q KKRYG.HG-- ---------- ---- 67
Ken05/Tk1 .LC-IWIKNN FSIGYIP.IY S.PQ.MTK.. ENQF.IRSGK .IQQ.QMMMF ITV.DINQ.Q KKRYG..G-- ---------- ---- 67
Kenya1950 .LC-IWIKNN FSIGYIP.IY S.LQ.MTK.. ENQF.IRSGK .IQQ.QMMMF ITG.DINQ.Q KKRYG..G-- ---------- ---- 67
Ken06.Bus .--------- ---------- ------TK.. ENQF.IRSGK .IQQ.QMMMF ITV.DINQ.Q KKRYG..G-- ---------- ---- 43
Conservation
