# Supplementary material for: A Retrospective Analysis Reveals That the 2021 Outbreaks of African Swine Fever Virus in Ghana Were Caused by Two Distinct Genotypes
Source: Viruses. 2024 Aug 7;16(8):1265. doi: 10.3390/v16081265 (PMC11360390; doi:10.3390/v16081265)
Supplement: Supplementary file 1 [file viruses-16-01265-s001.zip › Figure S1 Protein alignments.zip!/AlignmentPDF/E111R.pdf]

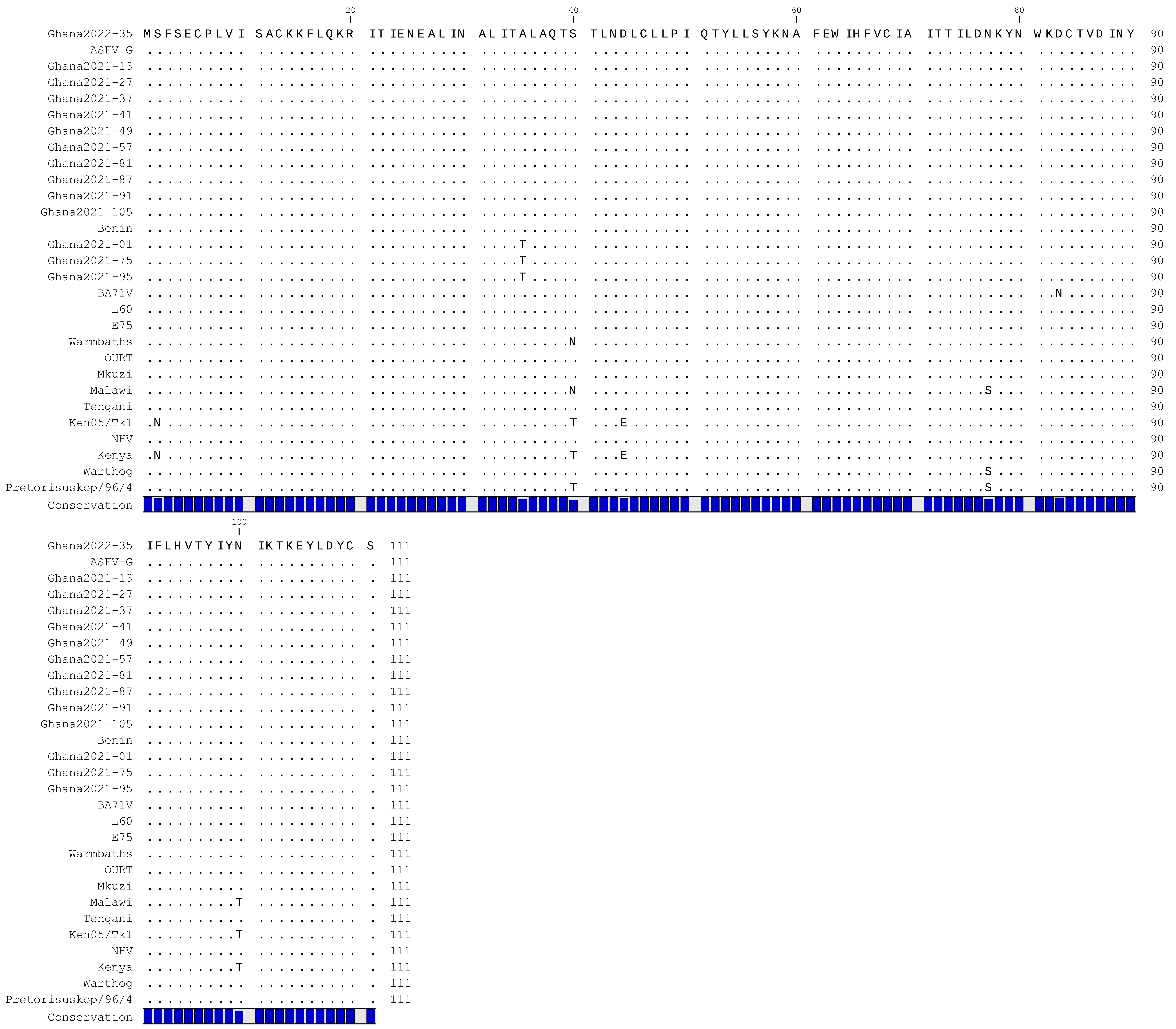
20 40 60 80
Ghana2022-35 MSFSECPLVI SACKKFLQKR ITIENEALIN ALITALAQTS TLNDLCLLPI QTYLLSYKNA FEWIHFVCIA ITTILDNKYN WKDCTVDINY 90
ASFV-G 90
Ghana2021-13 90
Ghana2021-27 90
Ghana2021-37 90
Ghana2021-41 90
Ghana2021-49 90
Ghana2021-57 90
Ghana2021-81 90
Ghana2021-87 90
Ghana2021-91 90
Ghana2021-105 90
Benin 90
Ghana2021-01 ....T..... 90
Ghana2021-75 ....T..... 90
Ghana2021-95 ....T..... 90
BA71V ....N....... 90
L60 90
E75 90
Warmbaths ....N 90
OURT 90
Mkuzi 90
Malawi ....N ....S... 90
Tengani 90
Ken05/Tk1 .N........ ....T ...E...... 90
NHV 90
Kenya .N........ ....T ...E...... 90
Warthog ....S... 90
Pretorisuskop/96/4 ....T ....S... 90
Conservation
100
Ghana2022-35 IFLHVTYIYN IKTKEYLDYC S 111
ASFV-G 111
Ghana2021-13 111
Ghana2021-27 111
Ghana2021-37 111
Ghana2021-41 111
Ghana2021-49 111
Ghana2021-57 111
Ghana2021-81 111
Ghana2021-87 111
Ghana2021-91 111
Ghana2021-105 111
Benin 111
Ghana2021-01 111
Ghana2021-75 111
Ghana2021-95 111
BA71V 111
L60 111
E75 111
Warmbaths 111
OURT 111
Mkuzi 111
Malawi ....T 111
Tengani 111
Ken05/Tk1 ....T 111
NHV 111
Kenya ....T 111
Warthog 111
Pretorisuskop/96/4 111
Conservation
