# Supplementary material for: A Retrospective Analysis Reveals That the 2021 Outbreaks of African Swine Fever Virus in Ghana Were Caused by Two Distinct Genotypes
Source: Viruses. 2024 Aug 7;16(8):1265. doi: 10.3390/v16081265 (PMC11360390; doi:10.3390/v16081265)
Supplement: Supplementary file 1 [file viruses-16-01265-s001.zip › Figure S1 Protein alignments.zip!/AlignmentPDF/E146L.pdf]

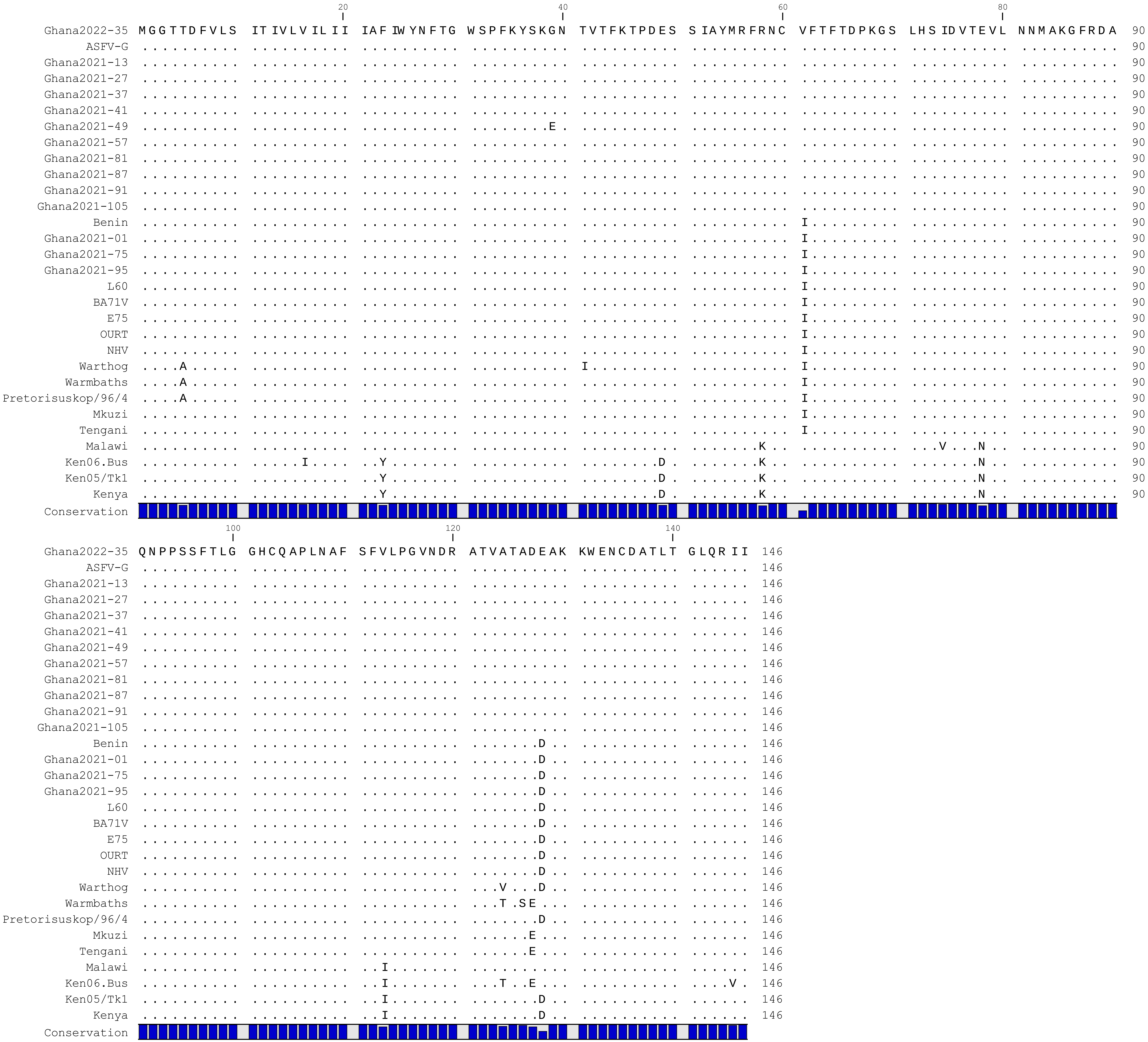
20
40
60
80
Ghana2022-35 MGGTTDFVLS ITIVLVILII IAFIWYNFTG WSPFKYSKGN TVTFKTPDES SIAYMRFRNC VFTFTDPKGS LHSIDVTEVL NNMAKGFRDA 90
100
120
140
Ghana2022-35 QNPPSSFTLG GHCQAPLNAF SFVLPGVNDR ATVATADEAK KWENCDATLT GLQRII 146
ASFV-G
Ghana2021-13
Ghana2021-27
Ghana2021-37
Ghana2021-41
Ghana2021-49
Ghana2021-57
Ghana2021-81
Ghana2021-87
Ghana2021-91
Ghana2021-105
Benin
Ghana2021-01
Ghana2021-75
Ghana2021-95
L60
BA71V
E75
OURT
NHV
Warthog
Warmbaths
Pretorisuskop/96/4
Mkuzi
Tengani
Malawi
Ken06.Bus
Ken05/Tk1
Kenya
Conservation
