# Supplementary material for: A Retrospective Analysis Reveals That the 2021 Outbreaks of African Swine Fever Virus in Ghana Were Caused by Two Distinct Genotypes
Source: Viruses. 2024 Aug 7;16(8):1265. doi: 10.3390/v16081265 (PMC11360390; doi:10.3390/v16081265)
Supplement: Supplementary file 1 [file viruses-16-01265-s001.zip › Figure S1 Protein alignments.zip!/AlignmentPDF/E183L.pdf]

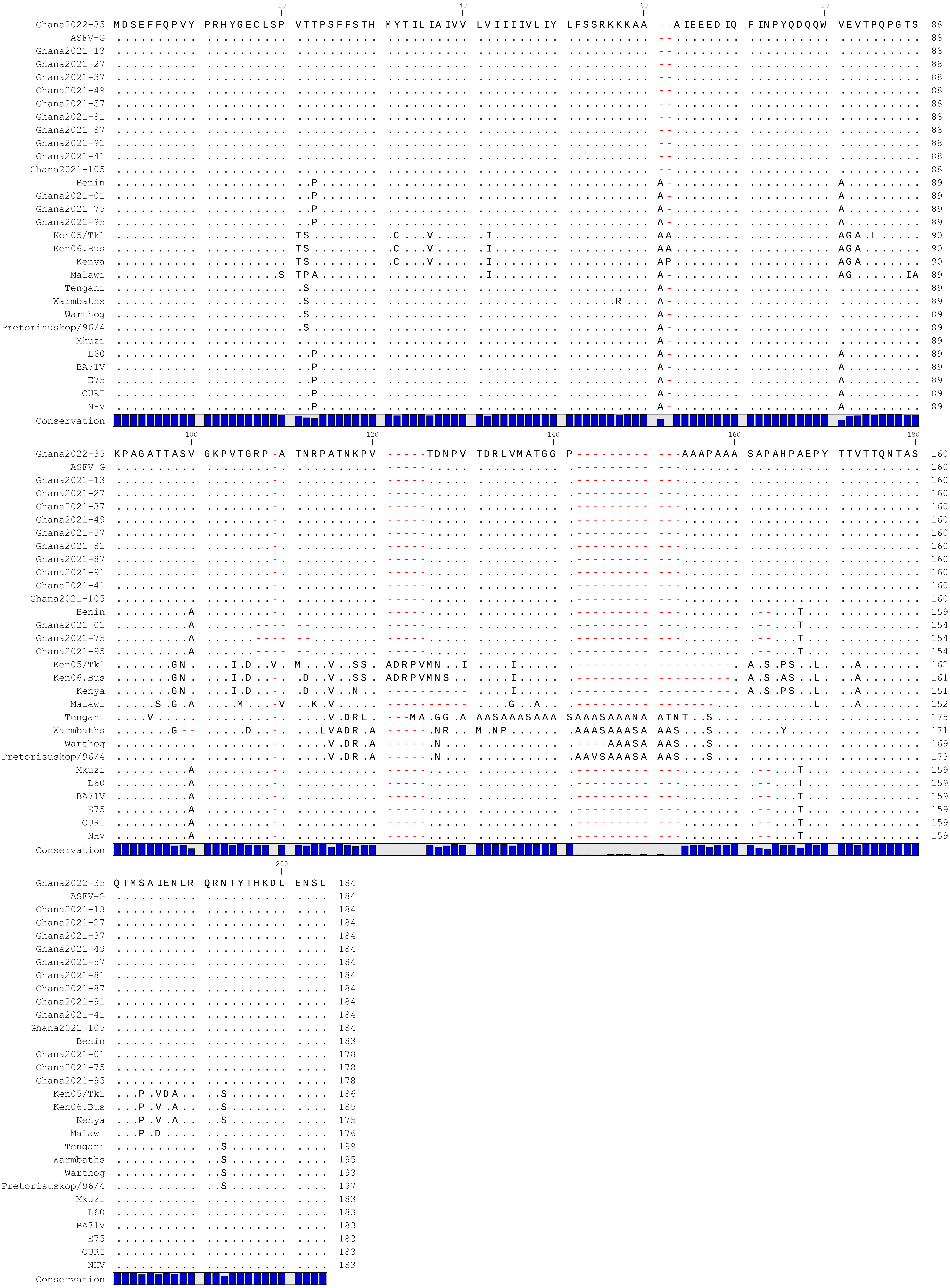

Ghana2022-35 MDSEFFQPVY PRHYGECLSP VTTPSFFSTH MYTILIAIVV LVIIIIVLIY LFSSRKKKAA --AIEEEDIQ FINPYQDQQW VEVTPQPGTS 88
Ghana2022-35 KPAGATTASV GKPVTGRP-A TNRPATNKPV -----TDNPV TDRLVMATGG P--------- ---AAAPAAA SAPAHPAEPY TTVTTQNTAS 160
Ghana2022-35 QTMSAIENLR QRNTYTHKDL ENSL 184
ASFV-G
Ghana2021-13
Ghana2021-27
Ghana2021-37
Ghana2021-49
Ghana2021-57
Ghana2021-81
Ghana2021-87
Ghana2021-91
Ghana2021-41
Ghana2021-105
Benin
Ghana2021-01
Ghana2021-75
Ghana2021-95
Ken05/Tk1
Ken06.Bus
Kenya
Malawi
Tengani
Warmbaths
Warthog
Pretorisuskop/96/4
Mkuzi
L60
BA71V
E75
OURT
NHV
Conservation
