# Supplementary material for: A Retrospective Analysis Reveals That the 2021 Outbreaks of African Swine Fever Virus in Ghana Were Caused by Two Distinct Genotypes
Source: Viruses. 2024 Aug 7;16(8):1265. doi: 10.3390/v16081265 (PMC11360390; doi:10.3390/v16081265)
Supplement: Supplementary file 1 [file viruses-16-01265-s001.zip › Figure S1 Protein alignments.zip!/AlignmentPDF/E199L.pdf]

```
                    20                                        40                                        60                                        80
Ghana2022-35        -MSCMPVSTK CNDIWVDFSC TGPSISELQK KEPKAWAAIL RSHTNQQTAE DDNIIGSICD KQGLCSKDEY AYSQYCACVN SGTLWPECAF 89
ASFV-G              -......... .......... .......... .......... .......... .......... .......... .......... .....A.... 89
Ghana2021-13        -......... .......... .......... .......... .......... .......... .......... .......... .....A.... 89
Ghana2021-27        -......... .......... .......... .......... .......... .......... .......... .......... .....A.... 89
Ghana2021-37        -......... .......... .......... .......... .......... .......... .......... .......... .....A.... 89
Ghana2021-41        -......... .......... .......... .......... .......... .......... .......... .......... .....A.... 89
Ghana2021-49        -......... .......... .......... .......... .......... .......... .......... .......... .....A.... 89
Ghana2021-57        -......... .......... .......... .......... .......... .......... .......... .......... .....A.... 89
Ghana2021-81        -......... .......... .......... .......... .......... .......... .......... .......... .....A.... 89
Ghana2021-87        -......... .......... .......... .......... .......... .......... .......... .......... .....A.... 89
Ghana2021-91        -......... .......... .......... .......... .......... .......... .......... .......... .....A.... 89
Ghana2021-105       -......... .......... .......... .......... .......... .......... .......... .......... .....A.... 89
Benin               -......... .......... .......... .......... .......... .......... .......... .......... .....A.... 89
Ghana2021-01        -......... .......... .......... .......... .......... .......... .......... .......... .....A.... 89
Ghana2021-75        -......... .......... I......... .......... .......... .......... .......... .......... .....A.... 89
Ghana2021-95        -......... .......... I......... .......... .......... .......... .......... .......... .....A.... 89
Malawi              -.....I... .......... ....L..... .......... ..QRS..... ..T....... .......N.. .......... .....A.... 89
Mkuzi               MS........ .......... ....V..... .......... .......... .......... .......... .......... .....A.... 90
BA71V               -......... .......... .......... .......... .......... .......... .......... .......... .....A.... 89
Tengani             -......... .......... .......... .......... .......... ..T....... .......... .......... .....A.... 89
OURT                -......... .......... .......... .......... .......... .......... .R........ .......... .....A.... 89
NHV                 -......... .......... .......... .......... .......... .......... .R........ .......... .....A.... 89
L60                 -......... .......... .......... .......... .......... .......... .......... .......... .....A.... 89
E75                 -......... .......... .......... .......... .......... .......... .......... .......... .....A.... 89
Warmbaths           -......... .......... .......... .......... ..R....... .......... .......... .......... .....A.... 89
Warthog             -......... .......... .......... .........V ..R....... .......... .......... .......... .....A.... 89
Pretorisuskop/96/4  -......... .......... .......... .......... .......... ..T....... .......... .......... .....A.... 89
Ken05/Tk1           -...T..... .....I.... .......... ...N...... ..QK...... ..T....... .......... .......... .....A.... 89
Kenya               -...T..... .....I.... ....V..... ...N...... ..QK...... ..T....... .......... .......... .....A.... 89
Ken06.Bus           -......... .......... .......... ...N.....S ..QK...... ..T....... .......... .......... .....A.... 89
Conservation

                             100                  120                  140                  160                  180
Ghana2022-35        APCNGNKNAY KTTEQRNILT NKQCPSGLTI CQNIAEYGGS GNISDLYQNF NCNSVINTFL INVMNHPFLT LILIILILII IYRLM-SSSG 178
ASFV-G              .......... .......... .......... .......... .......... .......... .......... .......... .....-.... 178
Ghana2021-13        .......... .......... .......... .......... .......... .......... .......... .......... .....-.... 178
Ghana2021-27        .......... .......... .......... .....V.... .......... .......... .......... .......... .....-.... 178
Ghana2021-37        .......... .......... .......... .......... .......... .......... .......... .......... .....-.... 178
Ghana2021-41        .......... .......... .......... .......... .......... .......... .......... .......... .....-.... 178
Ghana2021-49        .......... .......... .......... .......... .......... .......... .......... .......... .....-.... 178
Ghana2021-57        .......... .......... .......... .......... .......... .......... .......... .......... .....-.... 178
Ghana2021-81        .......... .......... .......... .......... .......... .......... .......... .......... .....-.... 178
Ghana2021-87        .......... .......... .......... .......R.. .......... .......... .......... .......... .....-.... 178
Ghana2021-91        .......... .......... .......... .......... .......... .......... .......... .......... .....-.... 178
Ghana2021-105       .......... .......... .......... .......... .......... .......... .......... .......... .....-.... 178
Benin               .......... .......... .......... .......E.. .......... .......... .......... .......... .....-P... 178
Ghana2021-01        .......... .......... .......... .......E.. .......... .......... .......... .......... .....-P... 178
Ghana2021-75        .......... .......... .......... .......E.. .......... .......... .......... .......... .....-P... 178
Ghana2021-95        .......... .......... .......... .......E.. .......... .......... .......... .......... .....-P... 178
Malawi              .......... ....H..... .......... .........T .......... .......... .......... ........V. .....-.... 178
Mkuzi               .......... .......... .......... .......R.. D......... .......... .......... .......... .....-.... 179
BA71V               .......... ....H..... .......... .......... .......... .......... .......... .......... .....-P... 178
Tengani             .......... ....H..... .......... .......... .......... .......... .......... .......... .....-.... 178
OURT                .......... .......... .......... .......R.. .......... .......... .......... .......... .....-P... 178
NHV                 .......... .......... .......... .......R.. .......... .......... .......... .......... .....-P... 178
L60                 .......... .......... .......... .......R.. .......... .......... .......... .......... .....-P... 178
E75                 .......... .......... .......... .......R.. .......... .......... .......... .......... .....-P... 178
Warmbaths           .......... .......... .......... .......... .......... .......... .......... .......... .....S.... 179
Warthog             .......... .......... .......... .......... .......... .......... .......... .......... .....S.... 179
Pretorisuskop/96/4  .......... .......... .......... .......... .......... .......... .......... .......... .....-.... 178
Ken05/Tk1           .......... .....K.... .......... .........T .......... .......... .......... ........V. .....-...A 178
Kenya               .......... .......... .......... .........T .......... .......... .......... ........V. .....-...A 178
Ken06.Bus           .......... ....H..... .......... .........T .......... .......... .......... ........V. .....-.... 178
Conservation

                              200
Ghana2022-35        GKHNDDKLPP PSLIFSNLNN F 199
ASFV-G              .......... .......... . 199
Ghana2021-13        .......... .......... . 199
Ghana2021-27        .......... .......... . 199
Ghana2021-37        .......... .......... . 199
Ghana2021-41        .......... .......... . 199
Ghana2021-49        .......... .......... . 199
Ghana2021-57        .......... .......... . 199
Ghana2021-81        .......... .......... . 199
Ghana2021-87        .......... .......... . 199
Ghana2021-91        .......... .......... . 199
Ghana2021-105       .......... .......... . 199
Benin               .......... .......... . 199
Ghana2021-01        .......... .......... . 199
Ghana2021-75        .......... .......... . 199
Ghana2021-95        .......... .......... . 199
Malawi              ....E..... .......... . 199
Mkuzi               .......... .......... L 200
BA71V               .......... .......... . 199
Tengani             ..P....... .......... . 199
OURT                .......... .......... . 199
NHV                 .......... .......... . 199
L60                 .......... .......... . 199
E75                 .......... .......... . 199
Warmbaths           .......... .......... . 200
Warthog             .......... .......... . 200
Pretorisuskop/96/4  .......... .......... . 199
Ken05/Tk1           A.Q.G..... .......... . 199
Kenya               A.Q.G..... .......... . 199
Ken06.Bus           A.Q.G..... .......... . 199
Conservation
```
