# Supplementary material for: A Retrospective Analysis Reveals That the 2021 Outbreaks of African Swine Fever Virus in Ghana Were Caused by Two Distinct Genotypes
Source: Viruses. 2024 Aug 7;16(8):1265. doi: 10.3390/v16081265 (PMC11360390; doi:10.3390/v16081265)
Supplement: Supplementary file 1 [file viruses-16-01265-s001.zip › Figure S1 Protein alignments.zip!/AlignmentPDF/E301R.pdf]

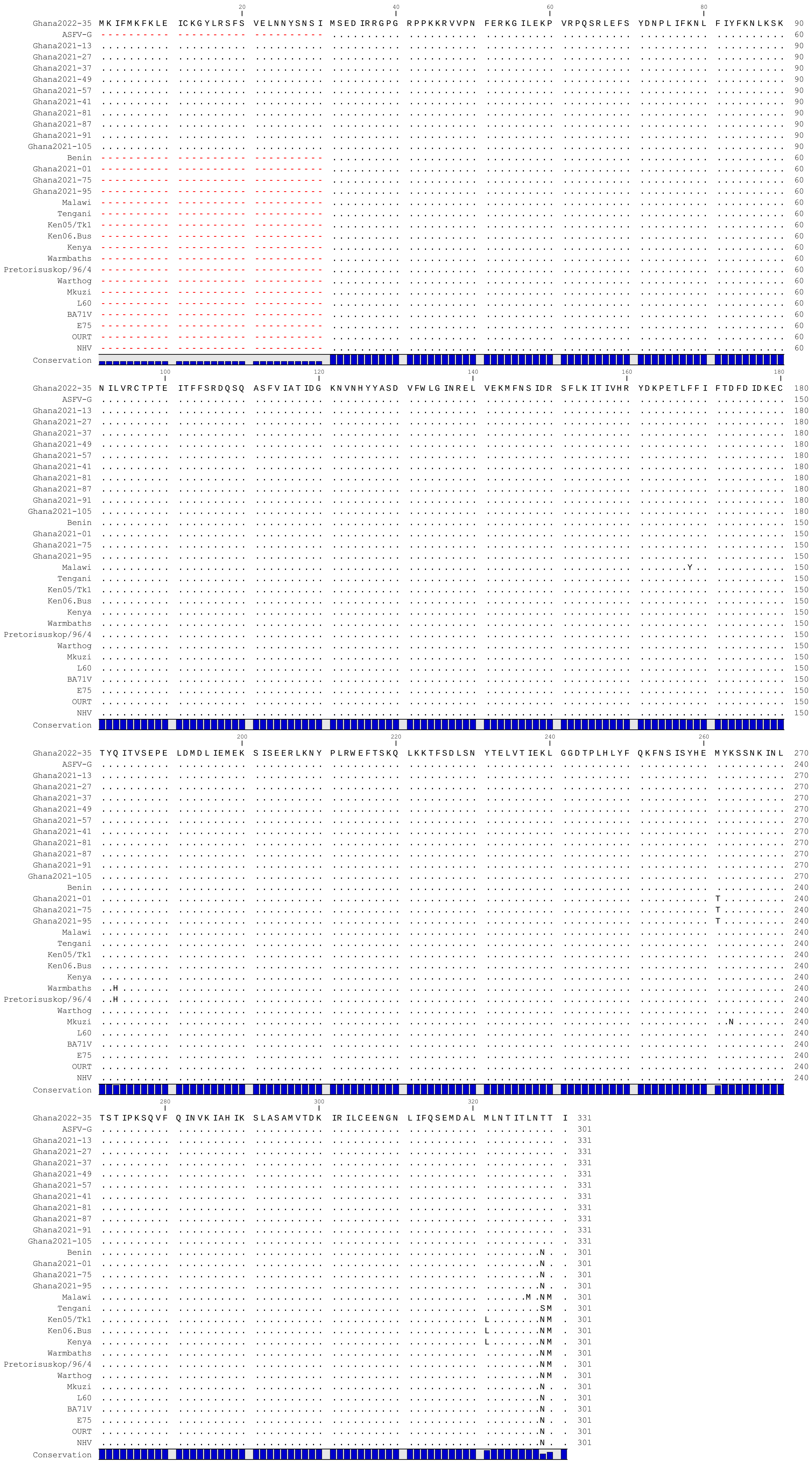

20 40 60 80
Ghana2022-35 MKIFMKFKLE ICKGYLRSFS VELNNYSNSI MSEDIRRGPG RPPKKRVVPN FERKGILEKP VRPQSRLEFS YDNPLIFKNL FIYFKNLKSK 90
ASFV-G ---------- ---------- ---------- 60
Ghana2021-13 90
Ghana2021-27 90
Ghana2021-37 90
Ghana2021-49 90
Ghana2021-57 90
Ghana2021-41 90
Ghana2021-81 90
Ghana2021-87 90
Ghana2021-91 90
Ghana2021-105 90
Benin ---------- ---------- ---------- 60
Ghana2021-01 ---------- ---------- ---------- 60
Ghana2021-75 ---------- ---------- ---------- 60
Ghana2021-95 ---------- ---------- ---------- 60
Malawi ---------- ---------- ---------- 60
Tengani ---------- ---------- ---------- 60
Ken05/Tk1 ---------- ---------- ---------- 60
Ken06.Bus ---------- ---------- ---------- 60
Kenya ---------- ---------- ---------- 60
Warmbaths ---------- ---------- ---------- 60
Pretorisuskop/96/4 ---------- ---------- ---------- 60
Warthog ---------- ---------- ---------- 60
Mkuzi ---------- ---------- ---------- 60
L60 ---------- ---------- ---------- 60
BA71V ---------- ---------- ---------- 60
E75 ---------- ---------- ---------- 60
OURT ---------- ---------- ---------- 60
NHV ---------- ---------- ---------- 60
Conservation
100 120 140 160 180
Ghana2022-35 NILVRCTPTE ITFFSRDQSQ ASFVIATIDG KNVNHYYASD VFWLGINREL VEKMFNSIDR SFLKITIVHR YDKPETLFFI FTDFDIDKEC 180
ASFV-G 150
Ghana2021-13 180
Ghana2021-27 180
Ghana2021-37 180
Ghana2021-49 180
Ghana2021-57 180
Ghana2021-41 180
Ghana2021-81 180
Ghana2021-87 180
Ghana2021-91 180
Ghana2021-105 180
Benin 150
Ghana2021-01 150
Ghana2021-75 150
Ghana2021-95 150
Malawi ....Y.. 150
Tengani 150
Ken05/Tk1 150
Ken06.Bus 150
Kenya 150
Warmbaths 150
Pretorisuskop/96/4 150
Warthog 150
Mkuzi 150
L60 150
BA71V 150
E75 150
OURT 150
NHV 150
Conservation
200 220 240 260
Ghana2022-35 TYQITVSEPE LDMDLIEMEK SISEERLKNY PLRWEFTSKQ LKKTFSDLSN YTELVTIEKL GGDTPLHLYF QKFNSISYHE MYKSSNKINL 270
ASFV-G 240
Ghana2021-13 270
Ghana2021-27 270
Ghana2021-37 270
Ghana2021-49 270
Ghana2021-57 270
Ghana2021-41 270
Ghana2021-81 270
Ghana2021-87 270
Ghana2021-91 270
Ghana2021-105 270
Benin 240
Ghana2021-01 .... T......... 240
Ghana2021-75 .... T......... 240
Ghana2021-95 .... T......... 240
Malawi 240
Tengani 240
Ken05/Tk1 240
Ken06.Bus 240
Kenya 240
Warmbaths ..H....... 240
Pretorisuskop/96/4 ..H....... 240
Warthog 240
Mkuzi ....N....... 240
L60 240
BA71V 240
E75 240
OURT 240
NHV 240
Conservation
280 300 320
Ghana2022-35 TSTIPKSQVF QINVKIAHIK SLASAMVTDK IRILCEENGN LIFQSEMDAL MLNTITLNTT I 331
ASFV-G 301
Ghana2021-13 331
Ghana2021-27 331
Ghana2021-37 331
Ghana2021-49 331
Ghana2021-57 331
Ghana2021-41 331
Ghana2021-81 331
Ghana2021-87 331
Ghana2021-91 331
Ghana2021-105 331
Benin ....N. . 301
Ghana2021-01 ....N. . 301
Ghana2021-75 ....N. . 301
Ghana2021-95 ....N. . 301
Malawi ....M.NM . 301
Tengani ....SM . 301
Ken05/Tk1 .... L.......NM . 301
Ken06.Bus .... L.......NM . 301
Kenya .... L.......NM . 301
Warmbaths ....NM . 301
Pretorisuskop/96/4 ....NM . 301
Warthog ....NM . 301
Mkuzi ....N. . 301
L60 ....N. . 301
BA71V ....N. . 301
E75 ....N. . 301
OURT ....N. . 301
NHV ....N. . 301
Conservation
