# Supplementary material for: A Retrospective Analysis Reveals That the 2021 Outbreaks of African Swine Fever Virus in Ghana Were Caused by Two Distinct Genotypes
Source: Viruses. 2024 Aug 7;16(8):1265. doi: 10.3390/v16081265 (PMC11360390; doi:10.3390/v16081265)
Supplement: Supplementary file 1 [file viruses-16-01265-s001.zip › Figure S1 Protein alignments.zip!/AlignmentPDF/EP364R.pdf]

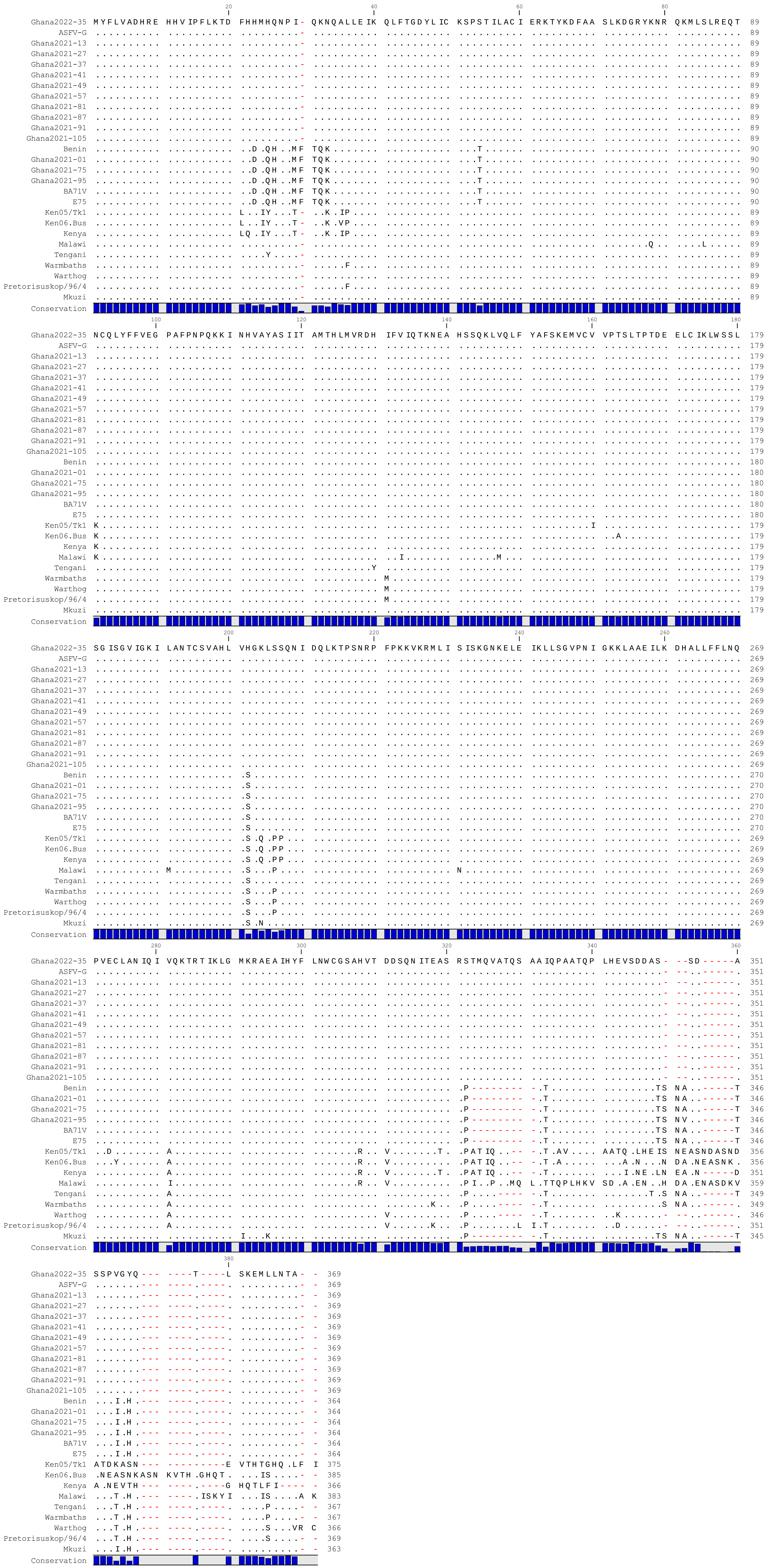
Ghana2022-35
ASFV-G
Ghana2021-13
Ghana2021-27
Ghana2021-37
Ghana2021-41
Ghana2021-49
Ghana2021-57
Ghana2021-81
Ghana2021-87
Ghana2021-91
Ghana2021-105
Benin
Ghana2021-01
Ghana2021-75
Ghana2021-95
BA71V
E75
Ken05/Tk1
Ken06.Bus
Kenya
Malawi
Tengani
Warmbaths
Warthog
Pretorisuskop/96/4
Mkuzi
Conservation
MYFLVADHRE HHVIPFLKTD FHHMHQNPI- QKNQALLEIK QLFTGDYLIC KSPSTILACI ERKTYKDFAA SLKDGRYKNR QKMLSLREQT 89
NCQLYFFVEG PAFPNPQKKI NHVAYASIIT AMTHLMVRDH IFVIQTKNEA HSSQKLVQLF YAFSKEMVCV VPTSLTPTDE ELCIKLWSSL 179
SGISGVIGKI LANTCSVAHL VHGKLSSQNI DQLKTPSNRP FPKKVKRMLI SISKGNKELE IKLLSGVPNI GKKLAAEILK DHALLFFLNQ 269
PVECLANIQI VQKTRTIKLG MKRAEAIHYF LNWCGSAHVT DDSQNITEAS RSTMQVATQS AAIQPAATQP LHEVSDDAS- --SD-----A 351
SSPVGYQ--- ----T----L SKEMLLNTA- - 369
