# Supplementary material for: A Retrospective Analysis Reveals That the 2021 Outbreaks of African Swine Fever Virus in Ghana Were Caused by Two Distinct Genotypes
Source: Viruses. 2024 Aug 7;16(8):1265. doi: 10.3390/v16081265 (PMC11360390; doi:10.3390/v16081265)
Supplement: Supplementary file 1 [file viruses-16-01265-s001.zip › Figure S1 Protein alignments.zip!/AlignmentPDF/K145R.pdf]

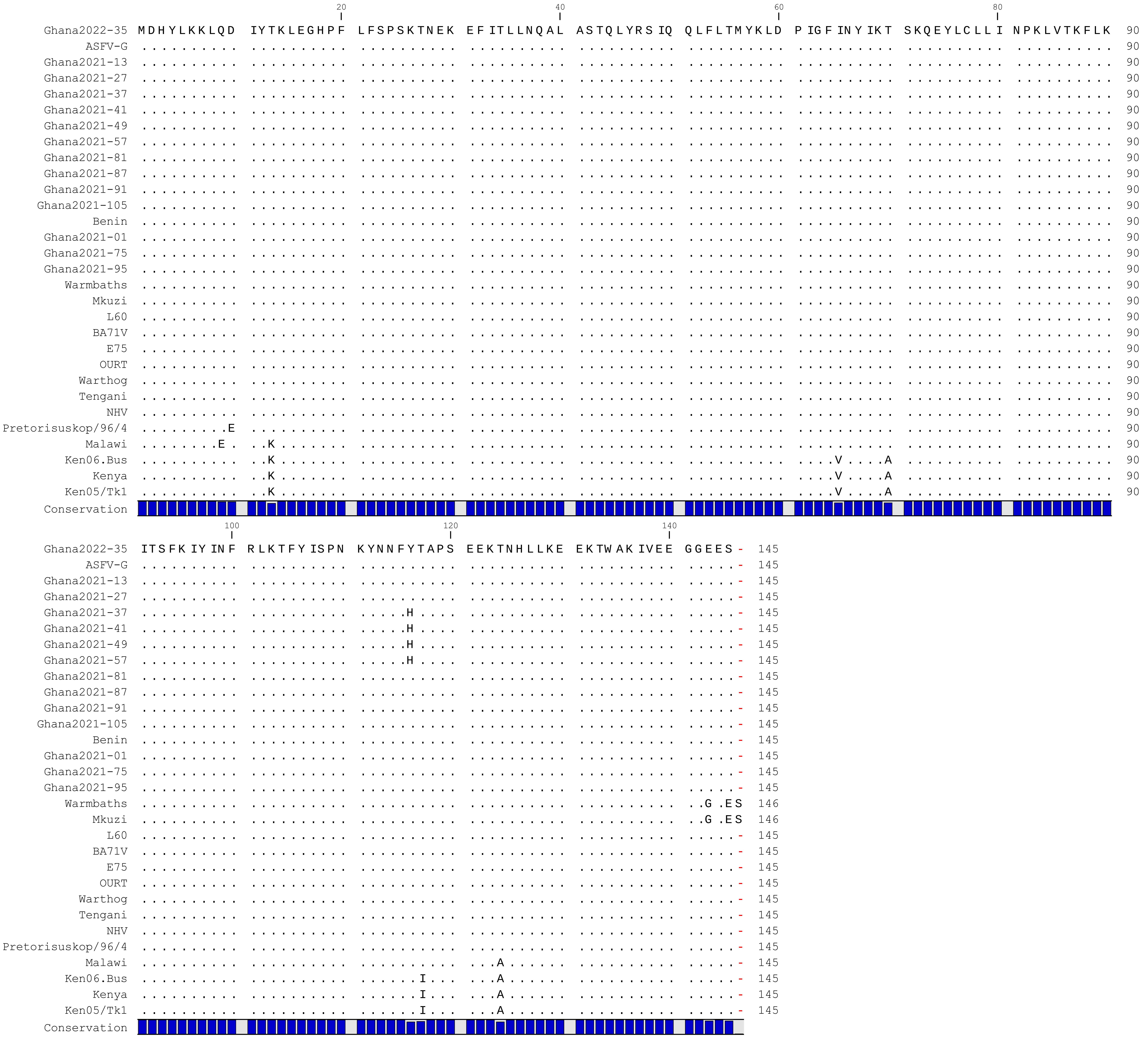

20 40 60 80
Ghana2022-35 MDHYLKKLQD IYTKLEGHPF LFSPSKTNEK EFITLLNQAL ASTQLYRSIQ QLFLTMYKLD PIGFINYIKT SKQEYLCLLI NPKLVTKFLK 90
ASFV-G .......... .......... .......... .......... .......... .......... .......... .......... .......... 90
Ghana2021-13 .......... .......... .......... .......... .......... .......... .......... .......... .......... 90
Ghana2021-27 .......... .......... .......... .......... .......... .......... .......... .......... .......... 90
Ghana2021-37 .......... .......... .......... .......... .......... .......... .......... .......... .......... 90
Ghana2021-41 .......... .......... .......... .......... .......... .......... .......... .......... .......... 90
Ghana2021-49 .......... .......... .......... .......... .......... .......... .......... .......... .......... 90
Ghana2021-57 .......... .......... .......... .......... .......... .......... .......... .......... .......... 90
Ghana2021-81 .......... .......... .......... .......... .......... .......... .......... .......... .......... 90
Ghana2021-87 .......... .......... .......... .......... .......... .......... .......... .......... .......... 90
Ghana2021-91 .......... .......... .......... .......... .......... .......... .......... .......... .......... 90
Ghana2021-105 .......... .......... .......... .......... .......... .......... .......... .......... .......... 90
Benin .......... .......... .......... .......... .......... .......... .......... .......... .......... 90
Ghana2021-01 .......... .......... .......... .......... .......... .......... .......... .......... .......... 90
Ghana2021-75 .......... .......... .......... .......... .......... .......... .......... .......... .......... 90
Ghana2021-95 .......... .......... .......... .......... .......... .......... .......... .......... .......... 90
Warmbaths .......... .......... .......... .......... .......... .......... .......... .......... .......... 90
Mkuzi .......... .......... .......... .......... .......... .......... .......... .......... .......... 90
L60 .......... .......... .......... .......... .......... .......... .......... .......... .......... 90
BA71V .......... .......... .......... .......... .......... .......... .......... .......... .......... 90
E75 .......... .......... .......... .......... .......... .......... .......... .......... .......... 90
OURT .......... .......... .......... .......... .......... .......... .......... .......... .......... 90
Warthog .......... .......... .......... .......... .......... .......... .......... .......... .......... 90
Tengani .......... .......... .......... .......... .......... .......... .......... .......... .......... 90
NHV .......... .......... .......... .......... .......... .......... .......... .......... .......... 90
Pretorisuskop/96/4 .........E .......... .......... .......... .......... .......... .......... .......... .......... 90
Malawi ........E. ..K....... .......... .......... .......... .......... .......... .......... .......... 90
Ken06.Bus .......... ..K....... .......... .......... .......... .......... ....V....A .......... .......... 90
Kenya .......... ..K....... .......... .......... .......... .......... ....V....A .......... .......... 90
Ken05/Tk1 .......... ..K....... .......... .......... .......... .......... ....V....A .......... .......... 90
Conservation
100 120 140
Ghana2022-35 ITSFKIYINF RLKTFYISPN KYNNFYTAPS EEKTNHLLKE EKTWAKIVEE GGEES- 145
ASFV-G .......... .......... .......... .......... .......... .....- 145
Ghana2021-13 .......... .......... .......... .......... .......... .....- 145
Ghana2021-27 .......... .......... .......... .......... .......... .....- 145
Ghana2021-37 .......... .......... .....H.... .......... .......... .....- 145
Ghana2021-41 .......... .......... .....H.... .......... .......... .....- 145
Ghana2021-49 .......... .......... .....H.... .......... .......... .....- 145
Ghana2021-57 .......... .......... .....H.... .......... .......... .....- 145
Ghana2021-81 .......... .......... .......... .......... .......... .....- 145
Ghana2021-87 .......... .......... .......... .......... .......... .....- 145
Ghana2021-91 .......... .......... .......... .......... .......... .....- 145
Ghana2021-105 .......... .......... .......... .......... .......... .....- 145
Benin .......... .......... .......... .......... .......... .....- 145
Ghana2021-01 .......... .......... .......... .......... .......... .....- 145
Ghana2021-75 .......... .......... .......... .......... .......... .....- 145
Ghana2021-95 .......... .......... .......... .......... .......... .....- 145
Warmbaths .......... .......... .......... .......... .......... ..G.ES 146
Mkuzi .......... .......... .......... .......... .......... ..G.ES 146
L60 .......... .......... .......... .......... .......... .....- 145
BA71V .......... .......... .......... .......... .......... .....- 145
E75 .......... .......... .......... .......... .......... .....- 145
OURT .......... .......... .......... .......... .......... .....- 145
Warthog .......... .......... .......... .......... .......... .....- 145
Tengani .......... .......... .......... .......... .......... .....- 145
NHV .......... .......... .......... .......... .......... .....- 145
Pretorisuskop/96/4 .......... .......... .......... .......... .......... .....- 145
Malawi .......... .......... .......... ...A...... .......... .....- 145
Ken06.Bus .......... .......... ......I... ...A...... .......... .....- 145
Kenya .......... .......... ......I... ...A...... .......... .....- 145
Ken05/Tk1 .......... .......... ......I... ...A...... .......... .....- 145
Conservation
