# Supplementary material for: A Retrospective Analysis Reveals That the 2021 Outbreaks of African Swine Fever Virus in Ghana Were Caused by Two Distinct Genotypes
Source: Viruses. 2024 Aug 7;16(8):1265. doi: 10.3390/v16081265 (PMC11360390; doi:10.3390/v16081265)
Supplement: Supplementary file 1 [file viruses-16-01265-s001.zip › Figure S1 Protein alignments.zip!/AlignmentPDF/MGF_360-14L.pdf]

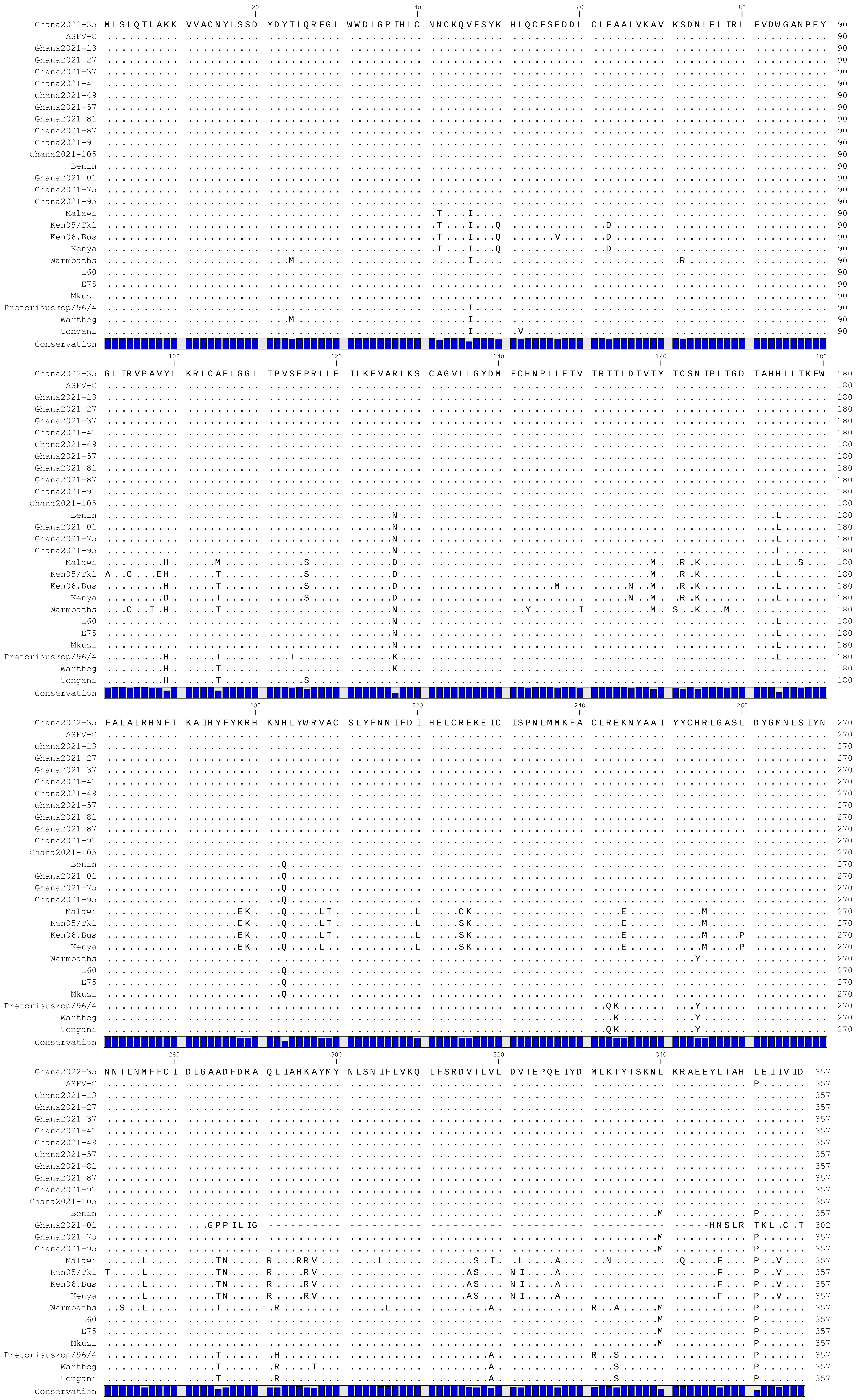

20 40 60 80
Ghana2022-35 MLSLQTLAKK VVACNYLSSD YDYTLQRFGL WWDLGPIHLC NNCKQVFSYK HLQCFSEDDL CLEAALVKAV KSDNLELIRL FVDWGANPEY 90
100 120 140 160 180
Ghana2022-35 GLIRVPAVYL KRLCAELGGL TPVSEPRLLE ILKEVARLKS CAGVLLGYDM FCHNPLLETV TRTTLDTVTY TCSNIPLTGD TAHHLLTKFW 180
200 220 240 260
Ghana2022-35 FALALRHNFT KAIHYFYKRH KNHLYWRVAC SLYFNNIFDI HELCREKEIC ISPNLMMKFA CLREKNYAAI YYCHRLGASL DYGMNLSIYN 270
280 300 320 340
Ghana2022-35 NNTLNMFFCI DLGAADFDRA QLIAHKAYMY NLSNIFLVKQ LFSRDVTLVL DVTEPQEIYD MLKTYTSKNL KRAEEYLTAH LEIIVID 357
ASFV-G
Ghana2021-13
Ghana2021-27
Ghana2021-37
Ghana2021-41
Ghana2021-49
Ghana2021-57
Ghana2021-81
Ghana2021-87
Ghana2021-91
Ghana2021-105
Benin
Ghana2021-01
Ghana2021-75
Ghana2021-95
Malawi
Ken05/Tk1
Ken06.Bus
Kenya
Warmbaths
L60
E75
Mkuzi
Pretorisuskop/96/4
Warthog
Tengani
Conservation
