# Supplementary material for: A Retrospective Analysis Reveals That the 2021 Outbreaks of African Swine Fever Virus in Ghana Were Caused by Two Distinct Genotypes
Source: Viruses. 2024 Aug 7;16(8):1265. doi: 10.3390/v16081265 (PMC11360390; doi:10.3390/v16081265)
Supplement: Supplementary file 1 [file viruses-16-01265-s001.zip › Figure S1 Protein alignments.zip!/AlignmentPDF/MGF_360-16R.pdf]

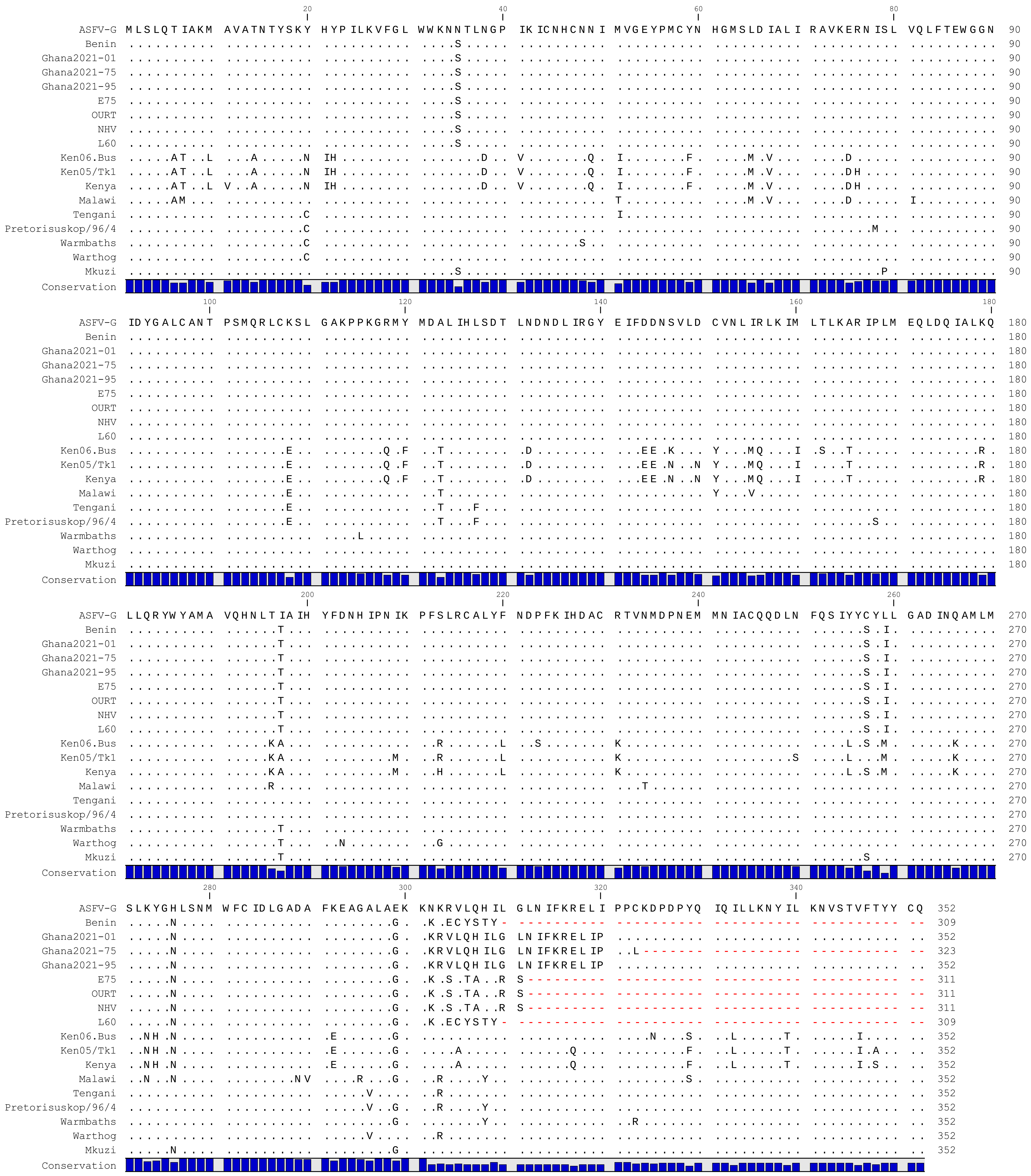

ASFV-G MLSLQTIAKM AVATNTYSKY HYPILKVFGL WWKNNTLNGP IKICNHCNNI MVGEYPMCYN HGMSLDIALI RAVKERNISL VQLFTEWGGN 90
ASFV-G IDYGALCANT PSMQRLCKSL GAKPPKGRMY MDALIHLSDT LNDNDLIRGY EIFDDNSVLD CVNLIRLKIM LTLKARIPLM EQLDQIALKQ 180
ASFV-G LLQRYWYAMA VQHNLTIAIH YFDNHIPNIK PFSLRCALYF NDPFKIHDAC RTVNMDPNEM MNIACQQDLN FQSIYYCYLL GADINQAMLM 270
ASFV-G SLKYGHLSNM WFCIDLGADA FKEAGALAEK KNKRVLQHIL GLNIFKRELI PPCKDPDPYQ IQILLKNYIL KNVSTVFTYY CQ 352
Benin
Ghana2021-01
Ghana2021-75
Ghana2021-95
E75
OURT
NHV
L60
Ken06.Bus
Ken05/Tk1
Kenya
Malawi
Tengani
Pretorisuskop/96/4
Warmbaths
Warthog
Mkuzi
Conservation
