# Supplementary material for: A Retrospective Analysis Reveals That the 2021 Outbreaks of African Swine Fever Virus in Ghana Were Caused by Two Distinct Genotypes
Source: Viruses. 2024 Aug 7;16(8):1265. doi: 10.3390/v16081265 (PMC11360390; doi:10.3390/v16081265)
Supplement: Supplementary file 1 [file viruses-16-01265-s001.zip › Figure S1 Protein alignments.zip!/AlignmentPDF/MGF_360-19R.pdf]

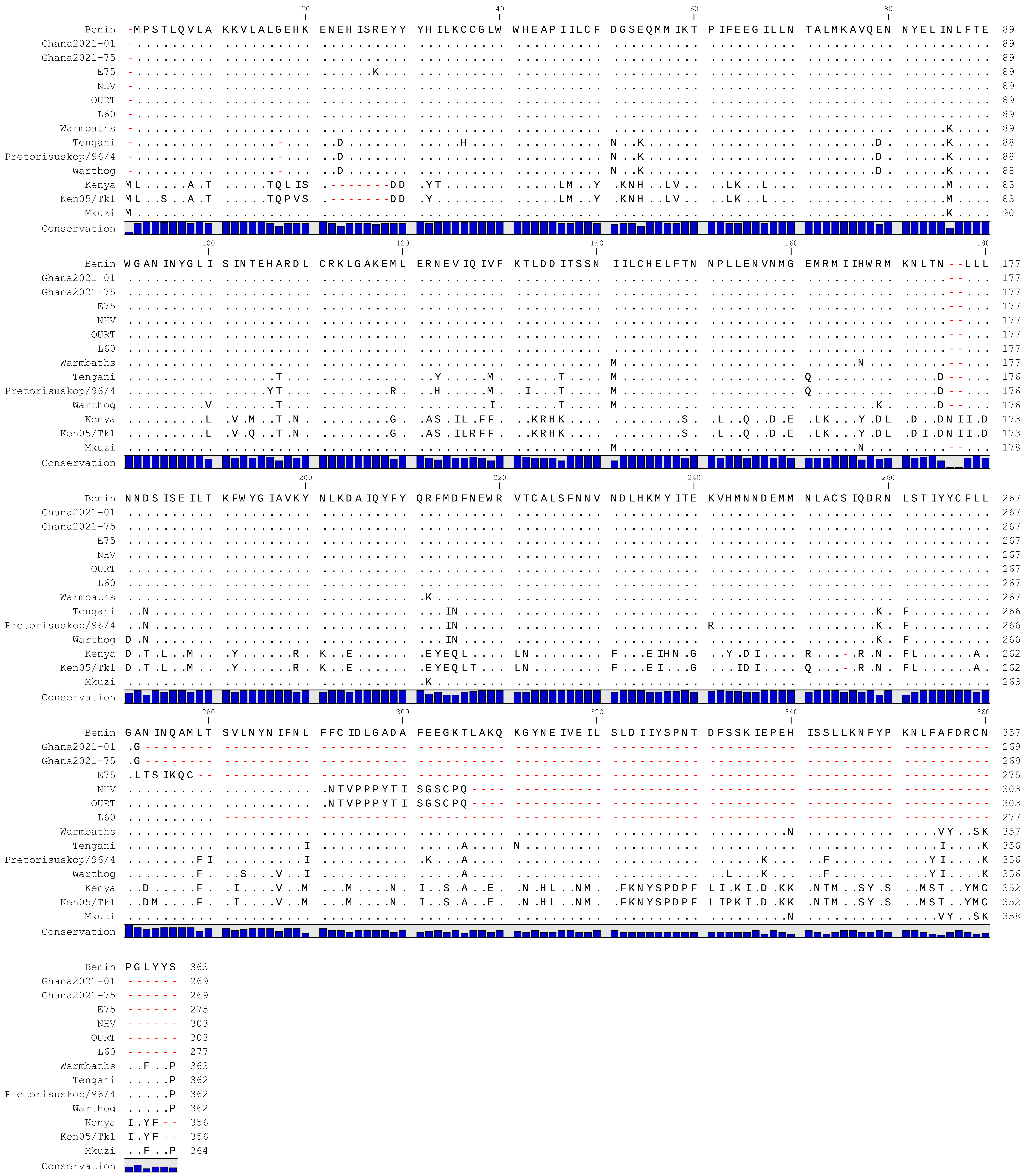

Benin
Ghana2021-01
Ghana2021-75
E75
NHV
OURT
L60
Warmbaths
Tengani
Pretorisuskop/96/4
Warthog
Kenya
Ken05/Tk1
Mkuzi
Conservation
-MPSTLQVLA KKVLALGEHK ENEHISREYY YHILKCCGLW WHEAPIILCF DGSEQMMIKT PIFEEGILLN TALMKAVQEN NYELINLFTE 89
WGANINYGLI SINTEHARDL CRKLGAKEML ERNEVIQIVF KTLDDITSSN IILCHELFTN NPLLENVNMG EMRMIIHWRM KNLTN--LLL 177
NNDSISEILT KFWYGIAVKY NLKDAIQYFY QRFMDFNEWR VTCALSFNNV NDLHKMYITE KVHMNNDEMM NLACSIQDRN LSTIYYCFLL 267
GANINQAMLT SVLNYNIFNL FFCIDLGADA FEEGKTLAKQ KGYNEIVEIL SLDIIYSPNT DFSSKIEPEH ISSLLKNFYP KNLFAFDRCN 357
PGLYYS 363
