# Supplementary material for: A Retrospective Analysis Reveals That the 2021 Outbreaks of African Swine Fever Virus in Ghana Were Caused by Two Distinct Genotypes
Source: Viruses. 2024 Aug 7;16(8):1265. doi: 10.3390/v16081265 (PMC11360390; doi:10.3390/v16081265)
Supplement: Supplementary file 1 [file viruses-16-01265-s001.zip › Figure S1 Protein alignments.zip!/AlignmentPDF/MGF_360-19Ra.pdf]

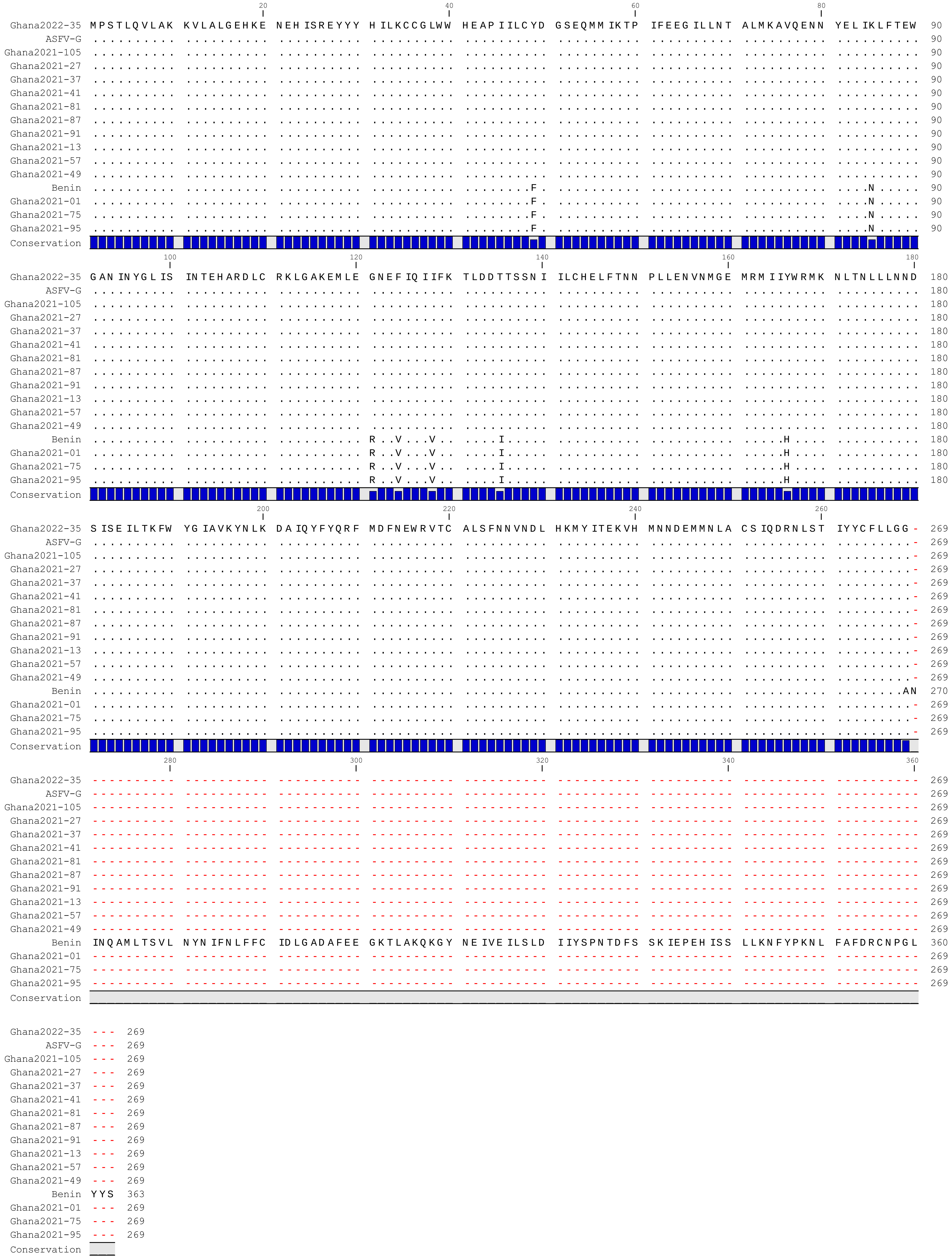

20 40 60 80
Ghana2022-35 MPSTLQVLAK KVLALGEHKE NEHISREYYY HILKCCGLWW HEAPIILCYD GSEQMMIKTP IFEEGILLNT ALMKAVQENN YELIKLFTEW 90
Benin .F .N 90
Ghana2021-01 .F .N 90
Ghana2021-75 .F .N 90
Ghana2021-95 .F .N 90
Conservation
100 120 140 160 180
Ghana2022-35 GANINYGLIS INTEHARDLC RKLGAKEMLE GNEFIQIIFK TLDDTTSSNI ILCHELFTNN PLLENVNMGE MRMIIYWRMK NLTNLLLNND 180
Benin R..V...V.. ....I..... .....H.... 180
Ghana2021-01 R..V...V.. ....I..... .....H.... 180
Ghana2021-75 R..V...V.. ....I..... .....H.... 180
Ghana2021-95 R..V...V.. ....I..... .....H.... 180
Conservation
200 220 240 260
Ghana2022-35 SISEILTKFW YGIAVKYNLK DAIQYFYQRF MDFNEWRVTC ALSFNNVNDL HKMYITEKVH MNNDEMMNLA CSIQDRNLST IYYCFLLGG- 269
Benin ....AN 270
Conservation
280 300 320 340 360
Benin INQAMLTSVL NYNIFNLFFC IDLGADAFEE GKTLAKQKGY NEIVEILSLD IIYSPNTDFS SKIEPEHISS LLKNFYPKNL FAFDRCNPGL 360
Conservation
Benin YYS 363
Conservation
