# Supplementary material for: A Retrospective Analysis Reveals That the 2021 Outbreaks of African Swine Fever Virus in Ghana Were Caused by Two Distinct Genotypes
Source: Viruses. 2024 Aug 7;16(8):1265. doi: 10.3390/v16081265 (PMC11360390; doi:10.3390/v16081265)
Supplement: Supplementary file 1 [file viruses-16-01265-s001.zip › Figure S1 Protein alignments.zip!/AlignmentPDF/MGF_360-1L.pdf]

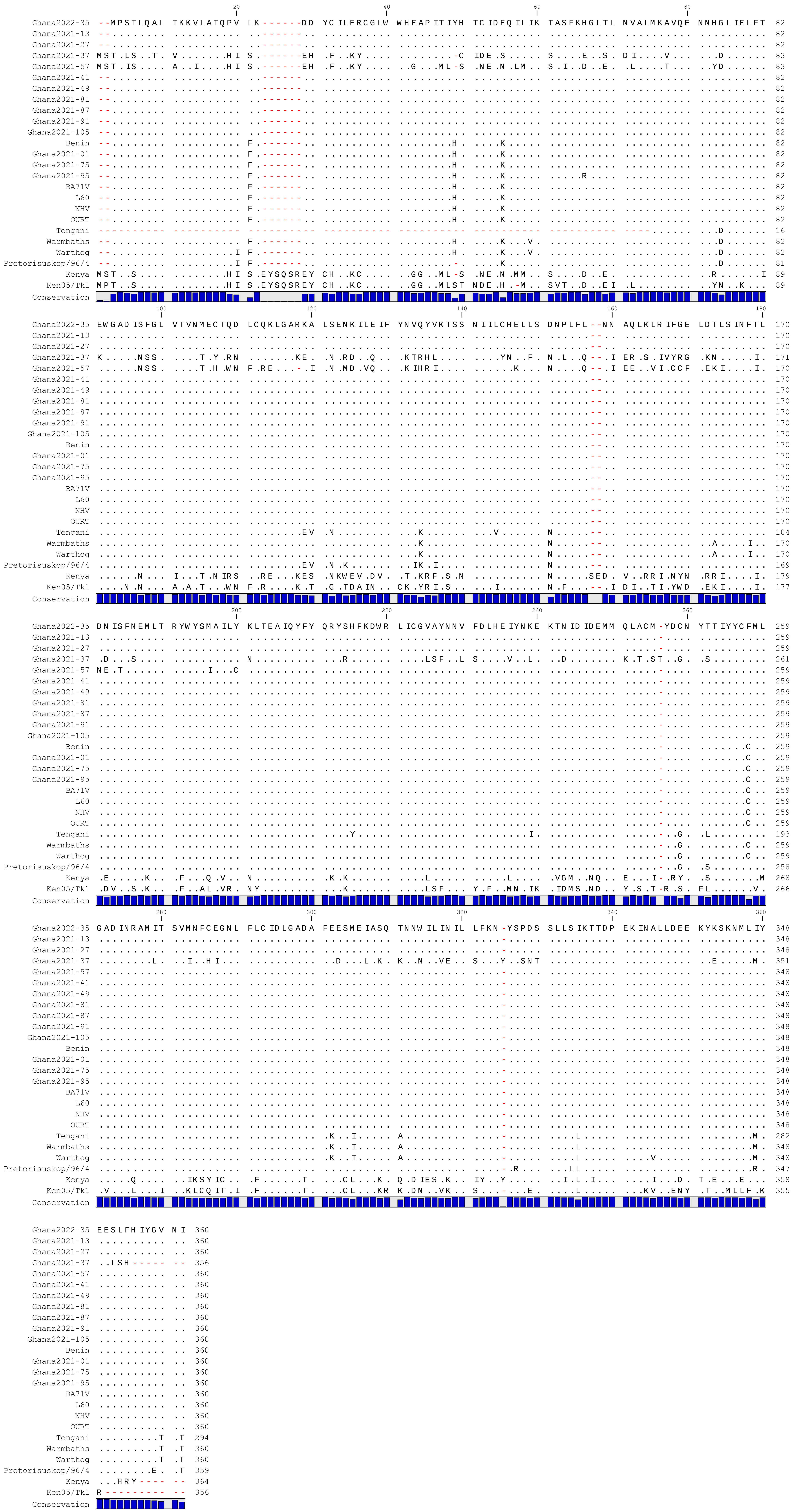

Ghana2022-35
Ghana2021-13
Ghana2021-27
Ghana2021-37
Ghana2021-57
Ghana2021-41
Ghana2021-49
Ghana2021-81
Ghana2021-87
Ghana2021-91
Ghana2021-105
Benin
Ghana2021-01
Ghana2021-75
Ghana2021-95
BA71V
L60
NHV
OURT
Tengani
Warmbaths
Warthog
Pretorisuskop/96/4
Kenya
Ken05/Tk1
Conservation
20
40
60
80
100
120
140
160
180
200
220
240
260
280
300
320
340
360
