# Supplementary material for: A Retrospective Analysis Reveals That the 2021 Outbreaks of African Swine Fever Virus in Ghana Were Caused by Two Distinct Genotypes
Source: Viruses. 2024 Aug 7;16(8):1265. doi: 10.3390/v16081265 (PMC11360390; doi:10.3390/v16081265)
Supplement: Supplementary file 1 [file viruses-16-01265-s001.zip › Figure S1 Protein alignments.zip!/AlignmentPDF/MGF_360-2L.pdf]

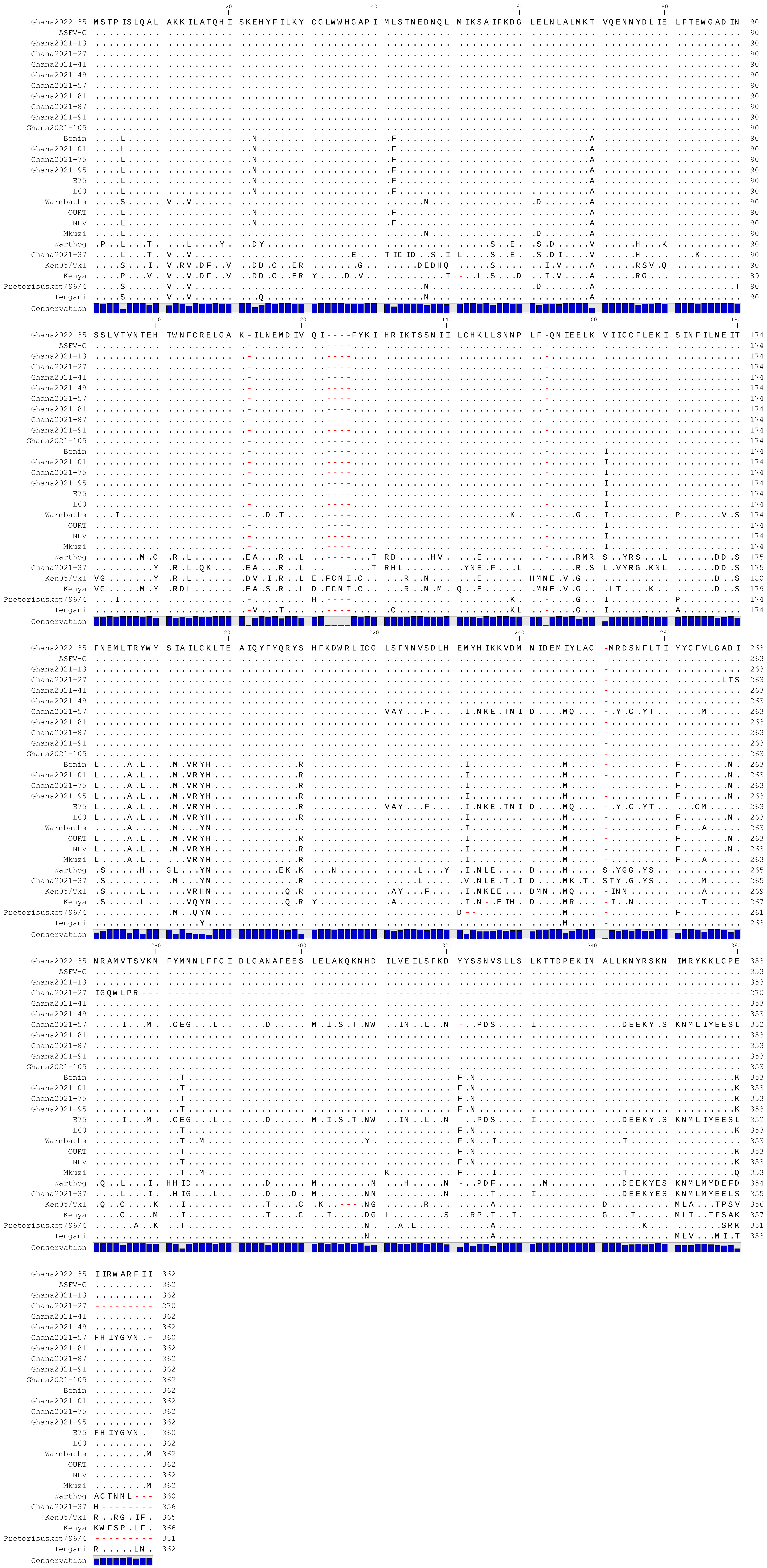

Ghana2022-35 MSTPISLQAL AKKILATQHI SKEHYFILKY CGLWWHGAPI MLSTNEDNQL MIKSAIFKDG LELNLALMKT VQENNYDLIE LFTEWGADIN 90
Ghana2022-35 SSLVTVNTEH TWNFCRELGA K-ILNEMDIV QI----FYKI HRIKTSSNII LCHKLLSNNP LF-QNIEELK VIICCFLEKI SINFILNEIT 174
Ghana2022-35 FNEMLTRYWY SIAILCKLTE AIQYFYQRYS HFKDWRLICG LSFNNVSDLH EMYHIKKVDM NIDEMIYLAC -MRDSNFLTI YYCFVLGADI 263
Ghana2022-35 NRAMVTSVKN FYMNNLFFCI DLGANAFEES LELAKQKNHD ILVEILSFKD YYSSNVSLLS LKTTDPEKIN ALLKNYRSKN IMRYKKLCPE 353
Ghana2022-35 IIRWARFII 362
ASFV-G
Ghana2021-13
Ghana2021-27
Ghana2021-41
Ghana2021-49
Ghana2021-57
Ghana2021-81
Ghana2021-87
Ghana2021-91
Ghana2021-105
Benin
Ghana2021-01
Ghana2021-75
Ghana2021-95
E75
L60
Warmbaths
OURT
NHV
Mkuzi
Warthog
Ghana2021-37
Ken05/Tk1
Kenya
Pretorisuskop/96/4
Tengani
Conservation
