# Supplementary material for: A Retrospective Analysis Reveals That the 2021 Outbreaks of African Swine Fever Virus in Ghana Were Caused by Two Distinct Genotypes
Source: Viruses. 2024 Aug 7;16(8):1265. doi: 10.3390/v16081265 (PMC11360390; doi:10.3390/v16081265)
Supplement: Supplementary file 1 [file viruses-16-01265-s001.zip › Figure S1 Protein alignments.zip!/AlignmentPDF/MGF_360-6L.pdf]

```
                                                    20                                      40                                      60                                      80
Ghana2022-35    MNSLQVLTKK VLIENKAFSN YHEDDIFILQ QLGLWWENGP IGFCKQCKMV TSGSMSCSDV DSYELDRALV RAVKKNQTDL IKLFVLWGAN  90
ASFV-G          .......... .......... .......... .......... .......... .......... .......... .......... ..........  90
Ghana2021-13    .......... .......... .......... .......... .......... .......... .......... .......... ..........  90
Ghana2021-27    .......... .......... .......... .......... .......... .......... .......... .......... ..........  90
Ghana2021-37    .......... .......... .......... .......... .......... .......... .......... .......... ..........  90
Ghana2021-41    .......... .......... .......... .......... .......... .......... .......... .......... ..........  90
Ghana2021-49    .......... .......... .......... .......... .......... .......... .......... .......... ..........  90
Ghana2021-57    .......... .......... .......... .......... .......... .......... .......... .......... ..........  90
Ghana2021-81    .......... .......... .......... .......... .......... .......... .......... .......... ..........  90
Ghana2021-87    .......... .......... .......... .......... .......... .......... .......... .......... ..........  90
Ghana2021-91    .......... .......... .......... .......... .......... .......... .......... .......... ..........  90
Ghana2021-105   .......... .......... .......... .......... .......... .......... .......... .......... ..........  90
Benin           .......... .........E .......... ......H... .......... .......... .......... .......... ..........  90
Ghana2021-01    .......... .........E .......... ......H... .......... .......... .......... .......... ..........  90
Ghana2021-75    .......... .........E .......... ......H... .......... .......... .......... .......... ..........  90
Ghana2021-95    .......... .........E .......... ......H... .......... .......... .......... .......... ..........  90
L60             .......... .........E .......... ......H... .......... .......... .......... .......... ..........  90
E75             .......... .........E .......... ......H... .......... .......... .......... .......... ..........  90
Kenya           .......... .......... .....S.... .......... .......... .G........ .......... K...E..... .........E  90
Ken05/Tk1       .......... ....T..... .......... ......HD.. .......... .......... .......... T......... ..........  90
Mkuzi           .......... .........E .......... ......H... .......... .......... .......... .......... ....I.....  90
Malawi          .......... .......... .....V.... ..E....... .......... .......... .......... K......... ..........  90
Conservation

                         100                                     120                                     140                                     160                                     180
Ghana2022-35    INYGIICAKT ERTKVLCIQL GADPKFLDVG LYNMFVDLIK QQKVLLAIDI YYDNISILDS FDSHDFYVLI DFIYNRFILN LDEKEKMIKN  180
ASFV-G          .......... .......... .......... .......... .......... .......... .......... .......... ..........  180
Ghana2021-13    .......... .......... .......... .......... .......... .......... .......... .......... ..........  180
Ghana2021-27    .......... .......... .......... .......... .......... .......... .......... .......... ..........  180
Ghana2021-37    .......... .......... .......... .......... .......... .......... .......... .......... ..........  180
Ghana2021-41    .......... .......... .......... .......... .......... .......... .......... .......... ..........  180
Ghana2021-49    .......... .......... .......... .......... .......... .......... .......... .......... ..........  180
Ghana2021-57    .......... .......... .......... .......... .......... .......... .......... .......... ..........  180
Ghana2021-81    .......... .......... .......... .......... .......... .......... .......... .......... ..........  180
Ghana2021-87    .......... .......... .......... .......... .......... .......... .......... .......... ..........  180
Ghana2021-91    .......... .......... .......... .......... .......... .......... .......... .......... ..........  180
Ghana2021-105   .......... .......... .......... .......... .......... .......... .......... .......... ..........  180
Benin           .......... ....D...E. ..N.E..... .......... .......... .......... .......... .....C.... ..........  180
Ghana2021-01    .......... ....D...E. ..N.E..... .......... .......... .......... .......... .....C.... ..........  180
Ghana2021-75    ...S...... ....D...E. ..N.E..... .......... .......... .......... .......... .....C.... ..........  180
Ghana2021-95    .......... ....D...E. ..N.E..... .......... .......... .......... .......... .....C.... ..........  180
L60             .......... ....D...E. ..N.E..... .......... .......... .......... .......... .....C.... ..........  180
E75             .......... ....D...E. ..N.E..... .......... .......... .......... .......... .....C.... ..........  180
Kenya           ..F..V.... .......... .......... .......... .......... .......... .......... .....G.... ......IT..  180
Ken05/Tk1       V....M.... .......L.. .......... ........L. .......... .......... ..D....T.. .......... .....E.TR.  180
Mkuzi           .......... .......... .......... ........L. .......... ....V..... .......... ..M......Y .....E.T..  180
Malawi          .........M .......... ....T..E.. ..T.....F. .......... ..E..P.... .......A.. N.....Y..Y .....E.TR.  180
Conservation

                                                   200                                     220                                     240                                     260
Ghana2022-35    TYVLKFWFKI AIEFNLIKPI RFLSKKFPHL DYWRLKTAVY LGNVDEIHHA YFQENIRLDP NDMMSLACMY PQNKLGIYYC FALGANINTA  270
ASFV-G          .......... .......... .......... .......... .......... .......... .......... .......... ..........  270
Ghana2021-13    .......... .......... .......... .......... .......... .......... .......... .......... ..........  270
Ghana2021-27    .......... .......... .......... .......... .......... .......... .......... .......... ..........  270
Ghana2021-37    .......... .......... .......... .......... .......... .......... .......... .......... ..........  270
Ghana2021-41    .......... .......... .......... .......... .......... .......... .......... .......... ..........  270
Ghana2021-49    .......... .......... .......... .......... .......... .......... .......... .......... ..........  270
Ghana2021-57    .......... .......... .......... .......... .......... .......... .......... .......... ..........  270
Ghana2021-81    .......... .......... .......... .......... .......... .......... .......... .......... ..........  270
Ghana2021-87    .......... .......... .......... .......... .......... .......... .......... .......... ..........  270
Ghana2021-91    .......... .......... .......... .......... .......... .......... .......... .......... ..........  270
Ghana2021-105   .......... .......... .......... .......... .......... .......... .......... .......... ..........  270
Benin           .......... .......... .......... .......... .......... .......... .......... .......... ..........  270
Ghana2021-01    .......... .......... .......... .......... .......... .......... .......... .......... ..........  270
Ghana2021-75    .......... .......... .......... .......... .......... .......... .......... .......... ..........  270
Ghana2021-95    .......... .......... .......... .......... .......... .......... .......... .......... ..........  270
L60             .......... .......... .......... .......... .......... .......... .......... .......... ..........  270
E75             .......... .......... .......... .......... .......... .......... .......... .......... ..........  270
Kenya           .L.....Y.F ......V... ...G...... .D........ .......... .......... .H........ .......... ..........  270
Ken05/Tk1       .L.....Y.F .......... .......... .H........ .......... .......... .N........ .......... ..........  270
Mkuzi           .L.......F ..Q....... .......... .D........ .......... .......... .......... .......... ..........  270
Malawi          .L........ .......... .......... .D....M... .......... .......... .H........ .......... ..........  270
Conservation

                         280                                     300                                     320                                     340                                     360
Ghana2022-35    LETLIGFINH EVNREITFFS NYGIWSNVHF CISLGANPYT KKIQETLLRQ EKNVMMKLLF KKGLLSPHSI RFTTNPLSS- ----------  349
ASFV-G          .......... .......... .......... .......... .......... .......... .......... LHKKILEP.E VRKIISTYEY  360
Ghana2021-13    .......... .......... .......... .......... .......... .......... .......... .........- ----------  349
Ghana2021-27    .......... .......... .......... .......... .......... .......... .......... .........- ----------  349
Ghana2021-37    .......... .......... .......... .......... .......... .......... .......... .........- ----------  349
Ghana2021-41    .......... .......... .......... .......... .......... .......... .......... .........- ----------  349
Ghana2021-49    .......... .......... .......... .......... .......... .......... .......... .........- ----------  349
Ghana2021-57    .......... .......... .......... .......... .......... .......... .......... .........- ----------  349
Ghana2021-81    .......... .......... .......... .......... .......... .......... .......... .........- ----------  349
Ghana2021-87    .......... .......... .......... .......... .......... .......... .......... .........- ----------  349
Ghana2021-91    .......... .......... .......... .......... .......... .......... .......... .........- ----------  349
Ghana2021-105   .......... .......... .......... .......... .......... .......... .......... .........- ----------  349
Benin           .......... .......... .......... .......... .......... ....I..... .......... LHKKILEP.E VRKIISTYEY  360
Ghana2021-01    .......... .......... .......... .......... .......... ....I..... .......... LHKKILEP.E VRKIISTYEY  360
Ghana2021-75    .......... .......... .......... .......... .......... ....I..... .......... LHKKILEP.E VRKIISTYEY  360
Ghana2021-95    .......... .......... .......... .......... .......... ....I..... .......... LHKKILEP.E VRKIISTYEY  360
L60             .......... .......... .......... .......... .......... ....I..... .......... LHKKILEP.E VRKIISTYEY  360
E75             .......... .......... .......... .......... .......... ....I..... .......... LHKKILEP.E VRKIISTYEY  360
Kenya           ..A..R.... .L..PF--.. .......... .......... .....I...E ..YDI..... .......... LHKKILEP.E VRKIISTYQY  358
Ken05/Tk1       .....R.... .......... .......... .......... .........E ....I..... .......... LHKKILEP.E VRKIISTYQY  360
Mkuzi           .......... .G........ .......... .......... .......... ....I..... .......... LHKKIIEPNE VRKIISTYEY  360
Malawi          .....R.... ..D....... .......... .........I .........E ....I...F. .....TS... LHKKILEPGE VRKIISTYEY  360
Conservation

Ghana2022-35    ---------- -----  349
ASFV-G          TETFHSFSSL RDNLR  375
Ghana2021-13    ---------- -----  349
Ghana2021-27    ---------- -----  349
Ghana2021-37    ---------- -----  349
Ghana2021-41    ---------- -----  349
Ghana2021-49    ---------- -----  349
Ghana2021-57    ---------- -----  349
Ghana2021-81    ---------- -----  349
Ghana2021-87    ---------- -----  349
Ghana2021-91    ---------- -----  349
Ghana2021-105   ---------- -----  349
Benin           TETFHSFSLL RDNLR  375
Ghana2021-01    TETFHSFSLL RDNLR  375
Ghana2021-75    TETFHSFSLL RDNLR  375
Ghana2021-95    TETFHSFSLL RDNLR  375
L60             TETFHSFSLL RDNLR  375
E75             TETFHSFSLL RDNLR  375
Kenya           TETFHSFSSL RDNVR  373
Ken05/Tk1       TETVHSFSSL RDNLR  375
Mkuzi           TETFHSFSLL RDNLQ  375
Malawi          TETFHSFSSL RDNLR  375
Conservation
```
