# Supplementary material for: A Retrospective Analysis Reveals That the 2021 Outbreaks of African Swine Fever Virus in Ghana Were Caused by Two Distinct Genotypes
Source: Viruses. 2024 Aug 7;16(8):1265. doi: 10.3390/v16081265 (PMC11360390; doi:10.3390/v16081265)
Supplement: Supplementary file 1 [file viruses-16-01265-s001.zip › Figure S1 Protein alignments.zip!/AlignmentPDF/MGF_505-1R.pdf]

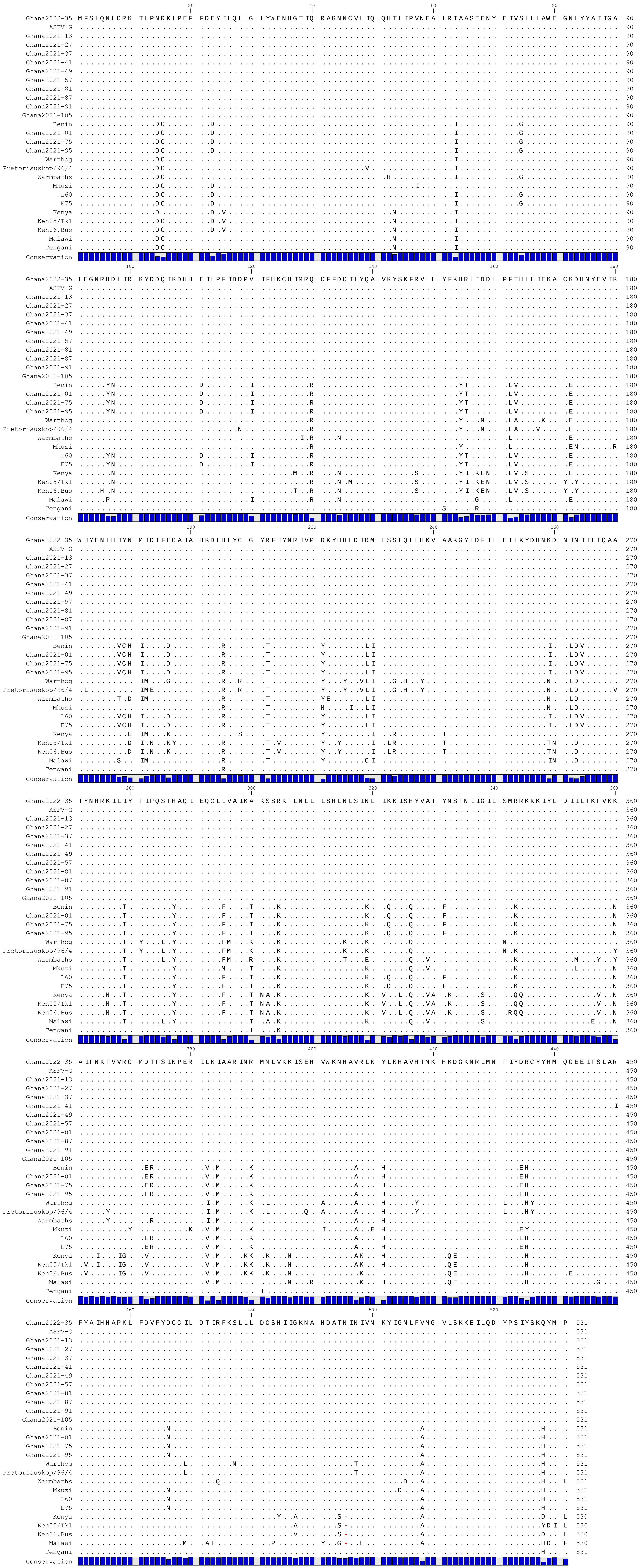
Ghana2022-35 MFSLQNLCRK TLPNRKLPEF FDEYILQLLG LYWENHGTIQ RAGNNCVLIQ QHTLIPVNEA LRTAASEENY EIVSLLLAWE GNLYYAIIGA 90
Ghana2022-35 LEGNRHDLIR KYDDQIKDHH EILPFIDDPV IFHKCHIMRQ CFFDCILYQA VKYSKFRVLL YFKHRLEDDL PFTHLLIEKA CKDHNYEVIK 180
Ghana2022-35 WIYENLHIYN MIDTFECAIA HKDLHLYCLG YRFIYNRIVP DKYHHLDIRM LSSLQLLHKV AAKGYLDFIL ETLKYDHNKD NINIILTQAA 270
Ghana2022-35 TYNHRKILIY FIPQSTHAQI EQCLLVAIKA KSSRKTLNLL LSHLNLSINL IKKISHYVAT YNSTNIIGIL SMRRKKKIYL DIILTKFVKK 360
Ghana2022-35 AIFNKFVVRC MDTFSINPER ILKIAARINR MMLVKKISEH VWKNHAVRLK YLKHAVHTMK HKDGKNRLMN FIYDRCYYHM QGEEIFSLAR 450
Ghana2022-35 FYAIHHAPKL FDVFYDCCIL DTIRFKSLLL DCSHIIGKNA HDATNINIVN KYIGNLFVMG VLSKKEILQD YPSIYSKQYM P 531
ASFV-G
Ghana2021-13
Ghana2021-27
Ghana2021-37
Ghana2021-41
Ghana2021-49
Ghana2021-57
Ghana2021-81
Ghana2021-87
Ghana2021-91
Ghana2021-105
Benin
Ghana2021-01
Ghana2021-75
Ghana2021-95
Warthog
Pretorisuskop/96/4
Warmbaths
Mkuzi
L60
E75
Kenya
Ken05/Tk1
Ken06.Bus
Malawi
Tengani
Conservation
