# Supplementary material for: A Retrospective Analysis Reveals That the 2021 Outbreaks of African Swine Fever Virus in Ghana Were Caused by Two Distinct Genotypes
Source: Viruses. 2024 Aug 7;16(8):1265. doi: 10.3390/v16081265 (PMC11360390; doi:10.3390/v16081265)
Supplement: Supplementary file 1 [file viruses-16-01265-s001.zip › Figure S1 Protein alignments.zip!/AlignmentPDF/MGF_505-9R.pdf]

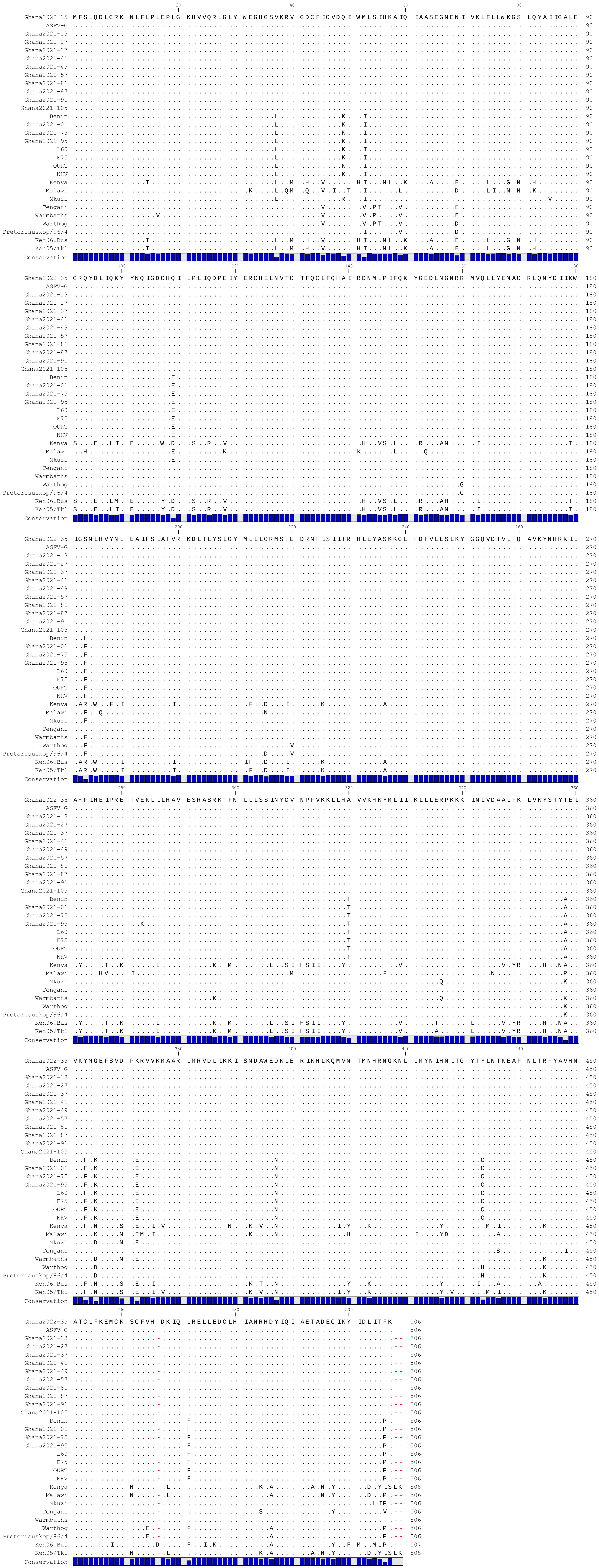

Ghana2022-35 MFSLQDLCRK NLFLPLEPLG KHVVQRLGLY WEGHGSVKRV GDCFICVDQI WMLSIHKAIQ IAASEGNENI VKLFLLWKGS LQYAIIGALE 90
Ghana2022-35 GRQYDLIQKY YNQIGDCHQI LPLIQDPEIY ERCHELNVTC TFQCLFQHAI RDNMLPIFQK YGEDLNGNRR MVQLLYEMAC RLQNYDIIKW 180
Ghana2022-35 IGSNLHVYNL EAIFSIAFVR KDLTLYSLGY MLLLGRMSTE DRNFISIITR HLEYASKKGL FDFVLESLKY GGQVDTVLFQ AVKYNHRKIL 270
Ghana2022-35 AHFIHEIPRE TVEKLILHAV ESRASRKTFR LLLSSINYCV NPFVKKLLHA VVKHKYMLII KLLLERPKKK INLVDAALFK LVKYSTYTEI 360
Ghana2022-35 VKYMGEFSVD PKRVVKMAAR LMRVDLIKKI SNDAWEDKLE RIKHLKQMVN TMNHRNGKNL LMYNIHNITG YTYLNTKEAF NLTRFYAVHN 450
Ghana2022-35 ATCLFKEMCK SCFVH-DKIQ LRELLEDCLH IANRHDYIQI AETADECIKY IDLITFK-- 506
ASFV-G
Ghana2021-13
Ghana2021-27
Ghana2021-37
Ghana2021-41
Ghana2021-49
Ghana2021-57
Ghana2021-81
Ghana2021-87
Ghana2021-91
Ghana2021-105
Benin
Ghana2021-01
Ghana2021-75
Ghana2021-95
L60
E75
OURT
NHV
Kenya
Malawi
Mkuzi
Tengani
Warmbaths
Warthog
Pretorisuskop/96/4
Ken06.Bus
Ken05/Tk1
Conservation
