# Supplementary material for: A Retrospective Analysis Reveals That the 2021 Outbreaks of African Swine Fever Virus in Ghana Were Caused by Two Distinct Genotypes
Source: Viruses. 2024 Aug 7;16(8):1265. doi: 10.3390/v16081265 (PMC11360390; doi:10.3390/v16081265)
Supplement: Supplementary file 1 [file viruses-16-01265-s001.zip › Figure S1 Protein alignments.zip!/AlignmentPDF/NP419L.pdf]

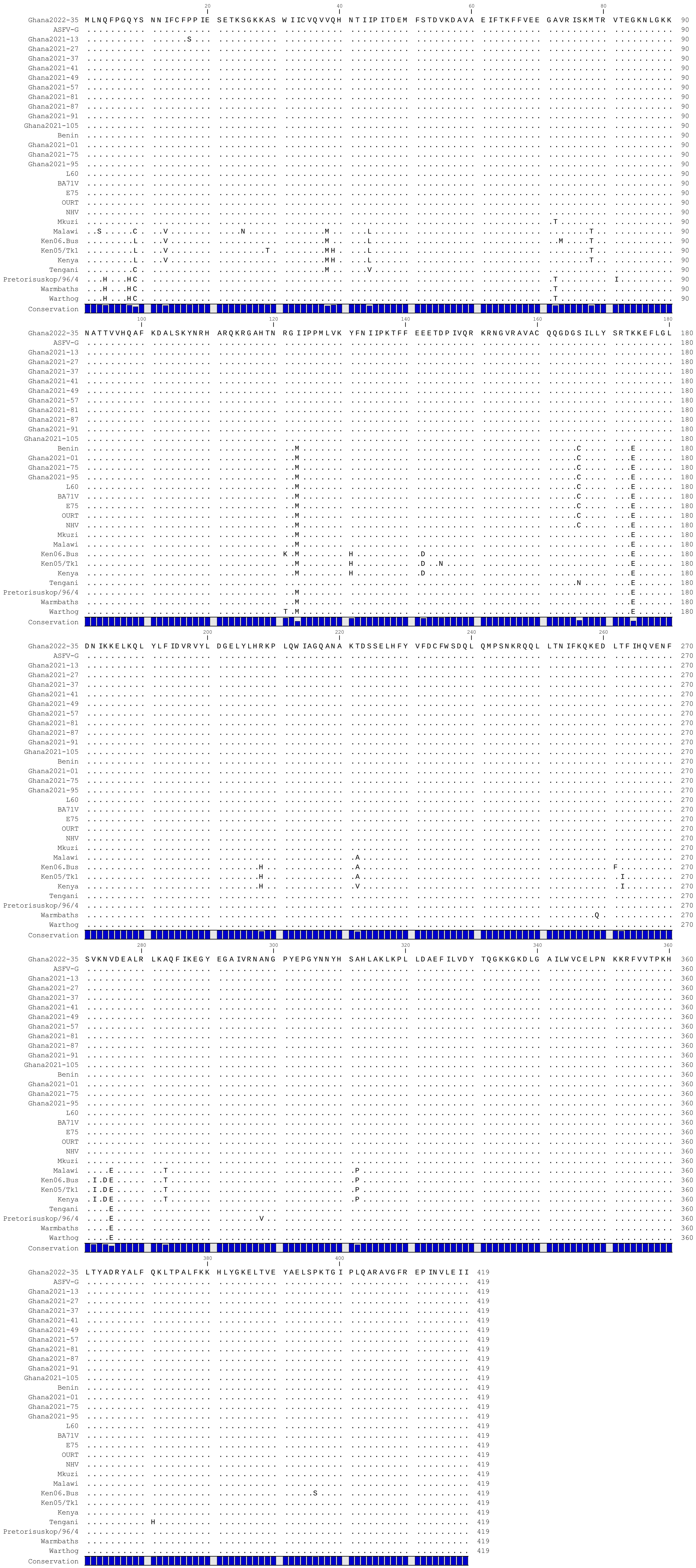

Ghana2022-35 MLNQFPGQYS NNIFCFPPIE SETKSGKKAS WIICVQVVQH NTIIPITDEM FSTDVKDAVA EIFTKFFVEE GAVRISKMTR VTEGKNLGKK 90
NATTVVHQAF KDALSKYNRH ARQKRGAHTN RGIIPPMLVK YFNIIPKTFF EEETDPIVQR KRNGVRAVAC QQGDGSILLY SRTKKEFLGL 180
DNIKKELKQL YLFIDVRVYL DGELYLHRKP LQWIAGQANA KTDSSELHFY VFDCFWSDQL QMPSNKRQQL LTNIFKQKED LTFIHQVENF 270
SVKNVDEALR LKAQFIKEGY EGAIVRNANG PYEPGYNNYH SAHLAKLKPL LDAEFILVDY TQGKKGKDLG AILWVCELPN KKRFVVTPKH 360
LTYADRYALF QKLTPALFKK HLYGKELTVE YAELSPKTGI PLQARAVGFR EPINVLEII 419
ASFV-G
Ghana2021-13
Ghana2021-27
Ghana2021-37
Ghana2021-41
Ghana2021-49
Ghana2021-57
Ghana2021-81
Ghana2021-87
Ghana2021-91
Ghana2021-105
Benin
Ghana2021-01
Ghana2021-75
Ghana2021-95
L60
BA71V
E75
OURT
NHV
Mkuzi
Malawi
Ken06.Bus
Ken05/Tk1
Kenya
Tengani
Pretorisuskop/96/4
Warmbaths
Warthog
Conservation
